# Tissue- and sex-specific lipidomic analysis of *Schistosoma mansoni* using high-resolution atmospheric pressure scanning microprobe matrix-assisted laser desorption/ionization mass spectrometry imaging

**DOI:** 10.1371/journal.pntd.0008145

**Published:** 2020-05-13

**Authors:** Patrik Kadesch, Thomas Quack, Stefanie Gerbig, Christoph G. Grevelding, Bernhard Spengler

**Affiliations:** 1 Institute of Inorganic and Analytical Chemistry, Justus Liebig University Giessen, Giessen, Germany; 2 Institute of Parasitology, Justus Liebig University Giessen, Biomedical Research Center Seltersberg (BFS), Giessen, Germany; The University of Melbourne, AUSTRALIA

## Abstract

Schistosomes are human pathogens causing the neglected tropical disease schistosomiasis, which occurs worldwide in (sub-)tropical regions. This infectious disease is often associated with poverty, and more than 700 million people are at risk of infection. Exploitation of novel habitats and limited therapeutic options brought schistosomes into research focus. Schistosomes are the only trematodes that have evolved separate sexes. They are covered by their metabolically active tegument, a surface area representing the interface between male and female in their permanent mating contact but also between parasite and host. The tegument comprises, besides others, numerous specific lipid compounds. Limited information is available on the exact lipid composition and its spatial distribution. We used atmospheric-pressure scanning microprobe matrix-assisted laser desorption/ionization (AP-SMALDI) mass spectrometry imaging (MSI) to characterize the *Schistosoma mansoni* tegument surface in comparison to tissue sections of whole worms or couples. We found that phosphatidylcholines (PC) and specific phosphatidylethanolamines (PE) are significantly more abundant inside the worm body compared to the tegument. On the other hand, the latter was found to be enriched in sphingomyelins (SM), phosphatidylserines (PS), lysophosphatidylcholines (LPC), and specific PE species. We further investigated lipid classes concerning number of carbon atoms in fatty acyl chains as well as the degree of unsaturation and found pronounced differences between the tegument and whole-worm body. Furthermore, differences between male and female teguments were found. The lipid composition of *S*. *mansoni* tissues has been investigated in an untargeted, spatially resolved manner for the first time.

## Author summary

WHO-defined Neglected Tropical Diseases, including schistosomiasis, are a burden for a significant part of the human world population. The fight against the diecious trematode *Schistosoma mansoni* can be supported by investigations of the specific molecular communication in male/female and in worm/host interactions. Improving the knowledge about *S*. *mansoni* is mandatory, since there is justified fear of the possibility of resistance development against the only available drug Praziquantel. We used mass spectrometry imaging as a powerful tool to provide topographic and tissue-specific information on the parasite. We investigated single male and female worms, as well as mating couples, regarding both, their inner tissue, and their intact surfaces, the tegument. We found highly specific lipid species and visualized their local distributions and abundances in high-resolution molecular images. Our findings may help to improve knowledge of the complex life cycles and of molecular communication mechanisms of schistosomes and may help to develop new drugs and strategies for treatment of the infectious disease.

## Introduction

Schistosomiasis is a waterborne disease with about 280 million people affected and many more at risk.[1] Untreated, multiple clinical manifestations with potential mortal outcome occur, counting for more than 200,000 deaths per year.[1] Especially in children, severe developmental impairments are observed, estimated to account for millions of disability-adjusted life years.[2] The disease is caused by pathogens of the genus *Schistosoma (S*. *haematobium*, *S*. *japonicum*, and *S*. *mansoni)*. The World Health Organization (WHO) has classified schistosomiasis as one of the neglected tropical diseases due to global spreading and its poverty-related endemicity.[1] With Praziquantel, there is currently only one widely applied drug available, and there is no vaccine yet.

Schistosomes exhibit a complex life cycle. When *S*. *mansoni* eggs are exposed to water, male and female miracidia hedge, which are infective for intermediate snail hosts of the genus *Biomphalaria*. Within the snail, asexual reproduction occurs, and multiple generations of sporocysts are produced that finally lead to the generation of *cercariae*, which are released to the aqueous environment, being infective for vertebrates such as humans. Upon skin penetration of the final host, *cercariae* lose their tails and transform into schistosomula, which follow the blood stream to the liver while they mature to the adult life-stage. After mating of males and females, the couples migrate to the mesenteric plexus of the gut (or the bladder in case of *S*. *haematobium*), where they shed approximately 300 eggs daily, many of which leave the body via feces (*S*. *japonicum*, *S*. *mansoni*) or urine (*S*. *haematobium*).

One of the biological peculiarities of schistosomes is that they have evolved separate sexes during evolution, whereas all other trematodes are hermaphrodites. Furthermore, a constant pairing contact is necessary for sexual maturation of the female, which is achieved by pairing-induced mitogenic activity and subsequent differentiation of the gonads. The latter consist of the ovary and the vitellarium.[3]

As trematodes, adult schistosomes exhibit characteristic organs such as oral sucker (OS), required for nutrient uptake, elimination of metabolites and pathogen-host interaction,[4] a ventral sucker (VS) for motility, the gut (GU), testes (TE; in males), ovary (OV) and vitellarium (VI) in females, and the tegument (T), the outer surface structure covering the worm body. The physiologically active tegument is unique to *Neodermata* and exhibits several key functions, crucial among others for nutrient uptake, host-parasite interaction, and survival.[5–10]

In the past couple of years, lipids have gained research interest not only as membrane components but also as functional constituents involved in cellular communication, key metabolic pathways, and as diagnostic markers.[11, 12] Therefore, various lipidomic studies have been conducted, contributing to our understanding of the characteristic lipid biology of schistosomes, especially in *S*. *mansoni*. For example, octadecenoic acid with a double bond position at Δ5, odd-chain fatty acyl phospholipid substituents, or long-chain fatty acids have been described.[13–18] Ceramides (Cer), known as signaling molecules, can be converted to sphingomyelins (SM) by addition of phosphocholine to the Cer hydroxyl head-group moiety.[19] By fluorescent lipid analogues, it has been demonstrated that SM of male schistosomes is synthesized from Cer, transported to the tegument and excreted to the medium.[19] Lipid extracts of whole-worms and tegument were analyzed by high performance liquid chromatography (HPLC), coupled to tandem mass spectrometry (MS), and it was shown that the tegument remarkably differs in phosphatidylserine (PS), phosphatidylcholine (PC), and lysophospholipid concentrations, compared to the inner part of the worm.[10] Great emphasis was placed on creating a comprehensive lipid atlas throughout all life stages of *S*. *mansoni*.[20] These data serve as the fundamental basis to investigate the functional role of specific lipids. The spatial information, which is lost during sample preparation for LC analysis, would strongly aid in understanding biological processes.

MALDI-MSI has been reported to be able to distinguish male and female based on individual *m*/*z*-signals.[21] However, discrimination was lacking when using unsupervised methods such as principle component analysis (PCA).[22] This was probably due to low spatial and mass resolution and/or topography-related signal intensity artifacts.

Mass spectrometry imaging (MSI)[23] adds valuable semi-quantitative, spatially resolved information to chemical investigations.[24] MSI is of special interest for untargeted metabolomics, because a wide variety of substance classes and valuable structural features can be assessed at once. One of the most advanced techniques with regard to spatial resolution is atmospheric pressure scanning microprobe matrix-assisted laser desorption/ionization (AP-SMALDI)[25] MSI. It is well suited for lipidomic analysis and capable to achieve high lateral resolution of 5–10 μm pixel size,[25] and 1.4 μm with an experimental setup, respectively.[26] When operating at such high spatial resolution on rough surfaces, topography-related artifacts may occur as the depth of focus is only about 40 μm at 5 μm laser focus diameter.[27] If not corrected, this leads to severe artifacts during the MALDI process, which can be overcome by autofocusing for each pixel. [27]

To investigate the spatial distribution of lipids in paired *S*. *mansoni* adults, AP-SMALDI MSI has been utilized to compare tegumental surface-associated lipid signals to those present inside the whole-worm body. Lipid signals were annotated based on a combination of Metaspace[28] data repository and a home-built, LC-MS/MS-based database. Elevated signal intensities were observed for sphingomyelins (SM), phosphatidylserines (PS), and lysophosphatidylcholins (LPC) at the tegument surface, as well as phosphatidylcholines (PC) and phosphatidylethanolamines (PE) for the worm body. Differentially abundant fatty acyl substituents were also found when comparing lipid composition of surfaces and sections. Furthermore, data analysis revealed differences between male and female surfaces, an aspect not yet addressed in previous studies on male-female interaction in the biochemical context.

## Methods

Quality grades and manufacturers of all chemicals used are shown in S1 Table.

### Ethic statement

Animal experiments were performed in accordance with the European Convention for the Protection of Vertebrate Animals used for experimental and other scientific purposes (ETS No 123; revised Appendix A) and were approved by the Regional Council (Regierungspraesidium) Giessen (V54-19 c 20/15 c GI 18/10).

### *Schistosoma mansoni* maintainance

Parasite maintenance was carried out as described elsewhere.[29] In brief, Syrian hamsters (*Mesocricetus auratus*) were infected with *cercariae* of *S*. *mansoni* (Liberian strain).[29] Worm couples were isolated 46 days post infection by hepatoportal perfusion.[30] Eggs were extracted from the livers of infected hamsters to obtain miracidia for infecting the intermediate snail host *Biomphalaria glabrata*.[29]

### Fixation and cryosectioning

Directly after perfusion, schistosome couples were separated by repetitive pipetting to obtain individual worms with known pairing history. The worm samples were prepared as described elsewhere.[31] In brief, worms were fixed in 6.7% glutaraldehyde solution in phosphate-buffered saline and snap-frozen in liquid nitrogen. Cryosections of couples were prepared at 30–40 μm thickness using a cryotome (HM 525, Thermo Fisher Scientific) after micro-embedding in 8% gelatin. Sections were mounted to glass slides (VWR, Radnor, PA, USA) and stored at -80°C until further analysis.

### Lipid extraction

Lipid extraction was carried out using a methyl-tert butylether (MtBE) protocol.[32] 200–500 worms were homogenized in 50 μL ice-cold ammonium acetate (50 mM) in a potter homogenizer (glass). 600 μL methyl-tert butylether (MtBE) and 150 μL methanol were added and shaken at 4°C for 1 h at 1,000 rpm (Thermomixer, Eppendorf, Hamburg, Germany). 200 μL water were added, shaken for 10 min (aforementioned conditions) and centrifuged at 1,000 x g for 5 min (Centrifuge 5804, Eppendorf). The organic phase was collected, and the aqueous phase was re-extracted with 400 μL MtBE, 120 μL methanol, and 100 μL water for 1 h (aforementioned conditions). After 5 min centrifugation at 1,000 x g, the organic phases were united, and the solvent was evaporated under a gentle stream of nitrogen in an ice bath until dryness. The dry extracts were dissolved in methanol:water 1:9 V/V to yield stock concentrations of 1 g of dried extract per mL and working solutions of 50 ng/μL.

### LC-MS analysis

Separation was conducted by ultra-high performance liquid chromatography (UHPLC; “Ultimate3000 RS”, Thermo Fisher Scientific, Bremen, Germany). The UHPLC was coupled to a “Q Exactive” orbital trapping mass spectrometer (Thermo Fisher Scientific, Bremen) via a heated electrospray ionization source (HESI II, Thermo Fisher Scientific) allowing data-dependent acquisition (DDA). The LC-MS method development was adapted from literature. [33] All parameters are shown in S2 Table.

### AP-SMALDI MS imaging

Intact male or female worms were taken from the freezer and fixed to glass slides by a thin film of gelatin; alternatively, cryosections of couples were used. Digital light microscopic images were recorded with a microscope (VHX5000, Keyence, Osaka, Japan) with 250- to 1000-fold magnification using a 3D-stitching mode. 2,5-dihydroxybenzoic acid (DHB, 30 mg/mL in acetone:water 1:1 V/V and 0.1% trifluoroacetic acid) was applied by pneumatic spraying of 70 μL of matrix solution at a flow rate of 10 μL/min, using a “SMALDIPrep” matrix preparation system (TransMIT GmbH, Giessen, Germany). The nebulizing nitrogen gas pressure was 1 bar. The sample was rotated with 500 rpm during pneumatic spraying.

For MS imaging, an autofocusing[27] “AP-SMALDI5 AF” ion source (TransMIT GmbH) was operated at 5 μm/pixel. 50 pulses/pixel were applied in positive-ion mode. An orbital trapping mass spectrometer (“Q Exactive HF”, Thermo Fisher Scientific, Bremen), was operated in scan mode from *m*/*z* 500–2000 at a mass resolution of 240,000 and a typical mass accuracy ≤ 3 ppm, using the lock-mass function (*m*/*z* 716.12462 = [5DHB-4H_2_O+NH_4_]^+^). The inlet capillary was heated to 250°C, and a potential of 3 kV was applied between sample holder and inlet capillary. This setup enabled molecular topographic analyses of complex surfaces with high accuracy in mass and space at scan rates of about 1 pixel/s. Image sizes are listed in Table 1.

**Table 1:** MSI image dimensions of acquired data files; 5 *μ*m/pixel, autofocusing mode, positive-ion polarity.

biological replicate number	male surface	female surface	mated couple section
**1**	280 x 280	400 x 174	340 x 300
**2**	400 x 300	235 x 275	290 x 265
**3**	420 x 270	300 x 260	300 x 272

### Data analysis

Data was converted into mzXML or MS2 file formats using Proteowizard[34] (v3.0.11028). The software packages LipidMatch[35] (v2.0.2) and Lipid Data Analyzer[36] (v2.6.2) were used for lipid identification based on fragmentation rules and retention time alignment, respectively. Only annotations found by both software tools were considered for database generation on lipid species level, specifying total fatty acyl chain lengths (or sphingosine base) and the number of double bonds. The data analysis process is visualized in S1 Fig. The database finally comprised triglycerides (TG), diglycerides (DG), sphingomyelins (SM), ceramides (Cer), phosphatidylcholines (PC), lyso-PC (LPC), phosphatidylethanolamines (PE), lyso-PE (LPE), phosphatidylserines (PS), phosphatidylglycerols (PG), and phosphatidylinositoles (PI) and included 469 lipid species in total.

MS imaging data were converted to imzML (imzML-converter v1.3[37]) and uploaded to Metaspace[28] for automated, FDR-controlled annotation using SwissLipids[38] database (data available through https://metaspace2020.eu/project/Kadesch-2019-Smansoni). Annotations were further processed manually for all measurements removing non-reproducible signals found in two or less samples (for graphical illustration see S2 Fig). Comparison with the aforementioned LC-MS database resulted in 227 lipids corresponding to 412 signals. Tissue regions were defined using region-of-interest (RoI) function in Mirion[39] (v3), exporting the mean, total ion current (TIC)-normalized signal intensities per RoI of aforementioned 412 *m*/*z*-signals (RoI was saved to imzML file). One RoI per MSI dataset was defined, to yield signal intensities for three biological replicates per class (worm body, male and female surfaces), enabling statistical analysis. For statistical analyses, the data matrix was imported to Perseus[40, 41] (v1.6.1.3), and the classification of lipid marker signals was done by hierarchical clustering (see step-by-step protocol in S3 Fig). MS ion images were visualized through MSiReader[42, 43] (v1) using the TIC normalization feature.

## Results and discussion

### Comparison of surface/tegument versus worm body tissue

Because of the uniqueness of the tegument, knowledge about its lipidome is of high interest. Only very few studies addressed the lipid composition of this highly specialized surface structure by MS.[9, 10, 21] Additionally, MSI studies have been conducted aiming to differentiate strains and sexes by MALDI-TOF (time of flight) MSI at low resolutions in both, mass and space, not ideally suited for untargeted metabolomics analysis.[21, 22] To shed further light onto the lipidome of the schistosome tegument, we performed AP-SMALDI MSI to characterize the surface area in comparison to the whole-body worm tissue at the molecular level. Since the tegument comprises an approximately 17 nm thick heptalaminate outer membrane,[44] it was not possible to discriminate different layers of the tegument and we therefore further refer to the term surface. Each worm was 5–10 mm long and less than 1 mm thick. The focal depth of the AP-SMALDI ion source at 5 μm lateral resolution is in the range of 40 μm, demanding for pixelwise laser focusing to obtain artifact-free MSI analysis.

**Fig 1:**
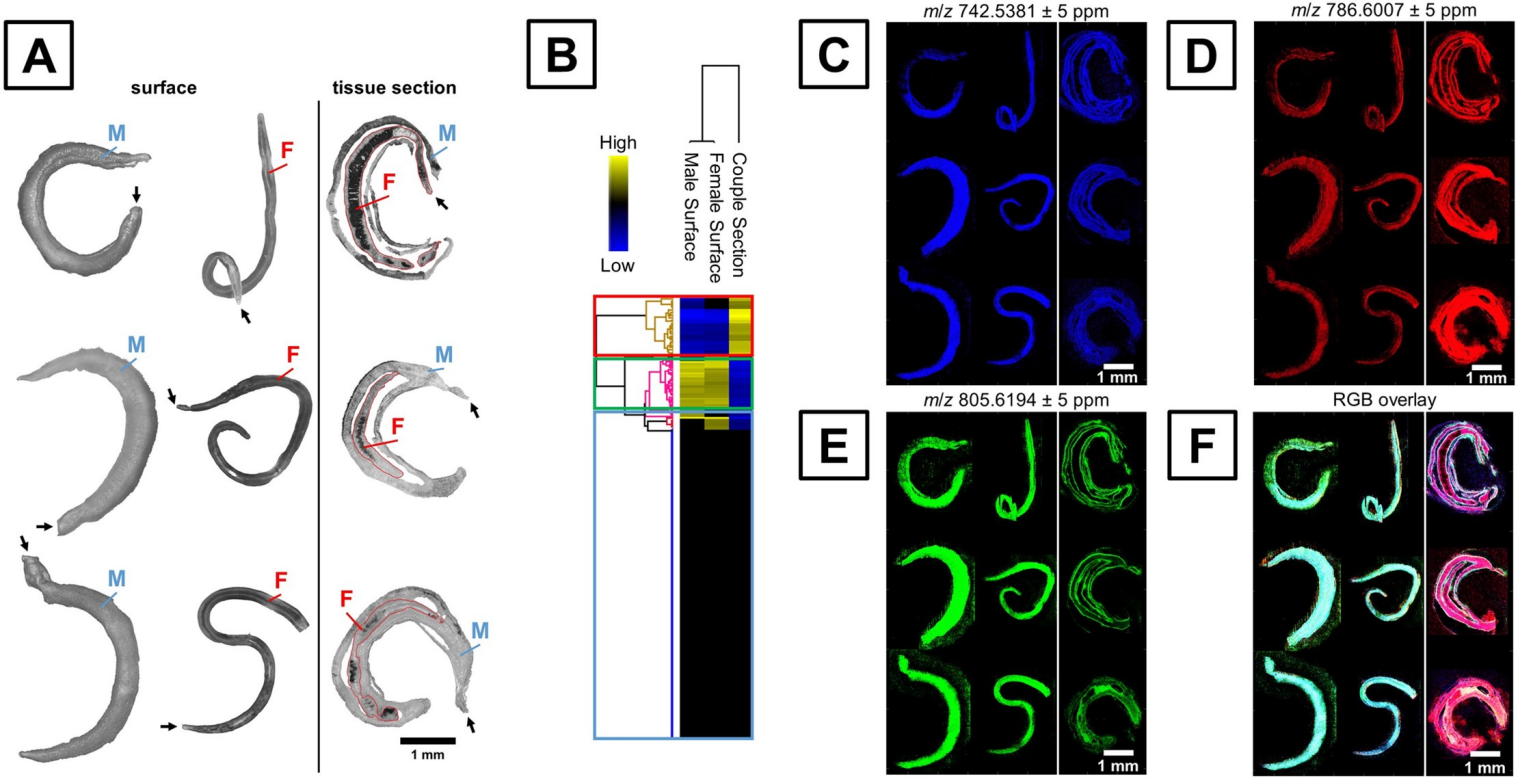
Results of MS imaging analysis conducted in positive-ion mode followed by multivariate statistical analysis. A–Light microscopic images of male (M) and female (F) *S*. *mansoni* surfaces and cryosections of couples recorded prior to matrix application. Black arrows indicate anterior end. All images are drawn to scale. B–Hierarchical clustering of mean TIC-normalized signal intensities indicating differentially abundant lipids on the surface versus inner tissue. Signals with elevated intensities in worm bodies are marked with a red box, prominent surface signals are boxed in green, and indifferent signals are indicated by a blue box. C–AP-SMALDI MS image of an indifferent signal at *m*/*z* 742.5381, equally distributed among surface and tissue, which was assigned to PE (36:3) as protonated molecule. D–MS image at *m*/*z* 786.6007 assigned to [PC (36:2) + H]^+^, showing increased signal intensities in the worm section. E–MS image at *m*/*z* 805.6194 assigned to SM (40:3) as sodiated ion, upregulated in the worm surface of males and females. F–Red-green-blue (RGB)-overlay of MS ion channels C to E, depicting the differences between surface and inner tissues. In total, the MS ion images comprised ~840,000 spectra, acquired with a spatial resolution of 5 μm in positive-ion mode.

Prior to data acquisition by MSI, microscope images were taken as shown in Fig 1A (more detailed in S4 Fig). Surface measurements of males (M) and females (F) are shown on the left side of Fig 1A and 1C–1F, while tissue sections of couples in biological triplicates are shown on the right side. The black arrow indicates the anterior end of the worms.

After mass spectrometric data acquisition, hierarchical clustering (HC) was performed based on relative signal intensities averaged over the whole tissue–containing area (Fig 1B). The signals shown in the HC can be separated in three major parts; specific signals for worm body tissue (red box) and surface (male and female; green box) and unspecific signals (blue box). Surface- or tissue-specific signals were obtained, although the number of unspecific signals predominated in this approach. The latter signals were classified as unspecific, because signal intensities varied between the biological replicates in excess of 5%, and/or insignificant differences occurred when comparing biological classes (surface vs tissue of adult worms). HC served as a ranking system for subsequent visualization of corresponding MS ion images by MSiReader (Fig 1C–1F), setting a mass deviation of ± 5 ppm for MS image generation.

Fig 1C shows an ion signal at *m*/*z* 742.5381 in blue, which was categorized unspecific by HC. The signal was assigned to protonated PE (36:3) and shows a homogenous distribution throughout all measurements and locations. The red ion channel in Fig 1D shows a signal, which is significantly upregulated inside the worm body. This signal was detected at *m*/*z* 786.6007 and was assigned to the protonated molecule of PC (36:2). Signals upregulated at the surface were also found as indicated by *m*/*z* 805.6194 in green (Fig 1E), which can be assigned to SM (40:3) as sodiated molecule. Additionally, the tegumental contours of the male and female worms can be observed in the cryosections by the same signal, confirming its higher abundance on the surface. The differential composition between surface and worm-body tissue becomes even more obvious in the red-green-blue overlay (RGB) (Fig 1F). While the whole worms are dominated by green colour, the sections are mostly red/purple. The classification approach by multivariate statistical analysis, particularly HC, was successfully used to classify *m*/*z*-signal intensities with respect to localized lipid compositions.

**Fig 2:**
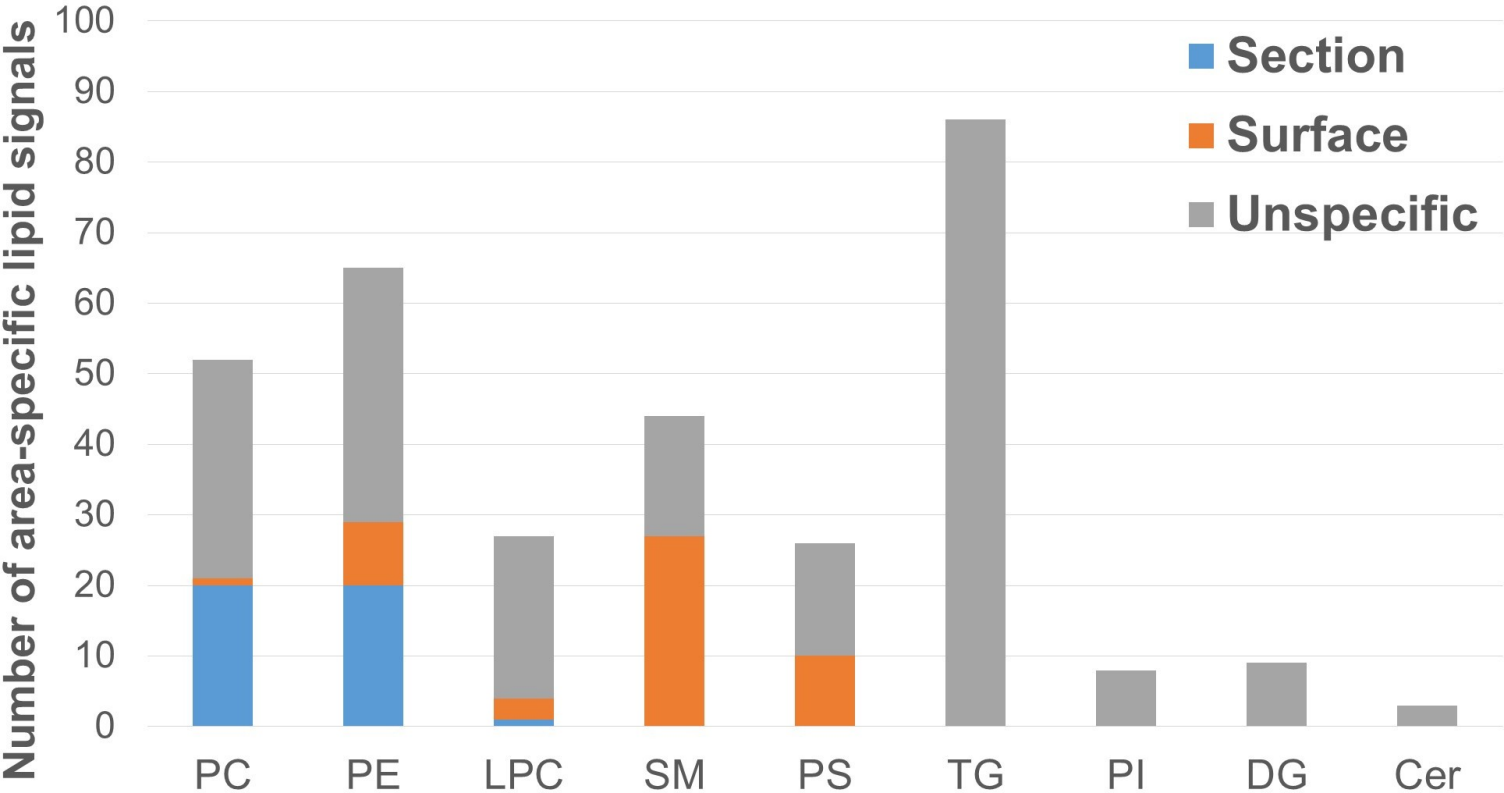
Location-specific lipids according to hierarchical clustering. Hierarchical clustering revealed specific lipid profiles inside the worm tissue (Section), on the worm surface (not discriminating between male and female) (Surface), and unspecific signals (either lacking intra-group reproducibility or significant inter-group differences) (Unspecific). Lipid sub-class abbreviations follow LipidMaps nomenclature.

Since ion images of individual lipid species are not representative for the abundance of the entire lipid class, we continued to investigate the signal intensities from worm surface and inner tissue for all lipid classes mentioned. Thereby, a number of marker signals were obtained being characteristic for either worm surface or inner tissue, not necessarily as the most abundant lipid species in the tissues, but rather signals of discrimination. Results are shown as Bar chart in **Fig 2**. A detailed description of all lipids summarized here can be found in S3 Table.

The extensive variation between surface and inner tissue can be recognized easily. The sum composition comprising all entities which passed analysis of variance (ANOVA) testing at a false-discovery-rate (FDR) of 5%, are shown in Fig 2A. Nine lipid classes are included in the diagram. TG, PE and PC formed the largest fractions.

Signals that were highly abundant inside the worm tissue are shown in Fig 2 (section). One significantly enriched species was a LPC, while all other marker signals exclusively belonged to the classes of PE and PC (20 substances per class). PE and PC lipid species can be isobaric and, therefore, are indiscriminable even at a high mass resolution of R = 240,000. However, using LC-MS/MS bulk analysis, we verified that both species were present in the worm although they were not distinguishable in MSI. PE and PC are common membrane constituents that determine membrane stability and rigidity.[45] Since the internal tissue of *S*. *mansoni* includes several tissue types and organs including the respective membranes, the observed predominance of PC and PE was expected. However, our findings are partially in contrast to previous publications which found a higher abundance of PC lipids in the tegument compared to the inner worm body by multiple-reaction-monitoring MS[10] and indirect quantitation by light scattering.[17] One explanation for this discrepancy might be that in our case, several isobaric lipids might have been analyzed as one signal. On the other hand, we did not take ion suppression effects into account, which can lead to altered signal intensity ratios compared to measurements with preceeding chromatographic separation. Two of the three major lipid constituents, indeed, show the same trend as described in previous publications (see S5 Fig). Another reason for disagreements is that tegument removal and lipid extraction are indirect methods, with intrinsic potential for artifact formation, compared to direct surface analysis by MSI.

A composition entirely different from the inner tissue was observed for the tegumental surface (Fig 2, in orange colour). The predominant class here was SM, besides PS and PE, and minor groups of LPC and PC. In comparison to the sum composition prior to HC, a relative specificity of SM and PS is obvious for the surface. It was reported previously, that SM accumulates at the *S*. *mansoni* surface where reversible breakdown to the respective ceramide lipids can occur.[19] The ceramides can enter the worm body freely while sphingomyelin is retained on the tegument.[19] Since these findings were derived from fluorescence microscopy, detailed information about individual lipid species was previously unavailable. Our findings enhance knowledge of SM species on the surface of schistosomes (see S3 Table). An increase in SM abundance is associated with increased membrane thickness and stability and has been well studied in a yeast model system.[45–47] However, the function of individual SM species remains unknown. PS have also been found in systematic studies of the tegument using a shotgun-lipidomics approach.[10] Our findings are well in accordance with this data. *S*. *mansoni*-derived PS could be of interest as they have been shown to act on toll-like receptor 2 (TLR2) in cell culture *in vitro*, in dependence of the fatty acyl substituents.[48] The binding pocket for PS on TLR2 might thus be a potential target for drug development. Minor signals of LPC and PC were found to be elevated on the surface. From literature it is known that *S*. *mansoni* has a rapid fatty acid turnover between phospholipid deacylation and reacylation.[17,18] Therefore, unraveling schistosomal PC acylases could be another topic in drug development.

In addition, signals with unspecific distribution were found in the tegument and in body tissue, showing no differential intensities within sample groups (Fig 2, unspecific). The lipid class composition of these unspecific lipids was similar to the sum composition. It is remarkable that although TG comprise the largest group of lipids before clustering, all TG lipids were classified as unspecific. However, some TGs were in fact specific for male vs female surfaces (see chapter below), and may have been removed during statistical testing, due to lower signal intensities and therefore more unstable signals compared to e.g. PC. Low pH value has been shown for schistosoma tissue. [49] It is therefore reasonable that PC is positively net-charged and readily detectable with high signal intensities under ambient conditions.

**Fig 3:**
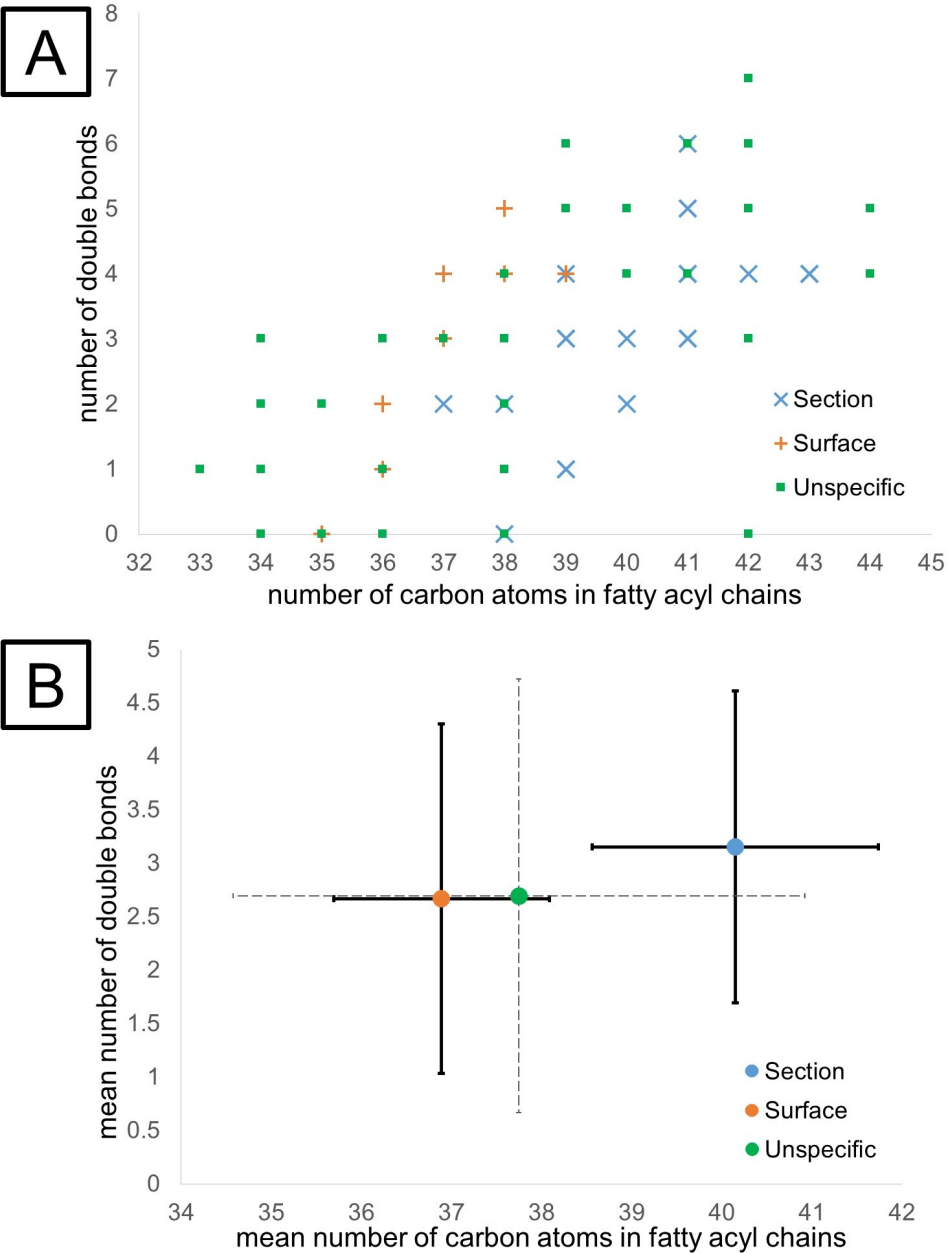
Number of carbon atoms in fatty acyl chains vs number of double bonds in detected phosphatidylethanolamines (PE). A–Worm tissue section-specific signals (blue cross), surface/tegument specific signals (orange +) and unspecific signals (green square). Overlapping indicators are attributed to the presence of several adducts corresponding to one lipid species. B–Arithmetic mean values of fatty acyl chain lengths and double bond numbers for worm tissue sections (blue), surface/tegument (orange) and unspecific signals (green). Error bars show the standard deviation across sections, surfaces and unspecific localizations, respectively.

PEs appeared as marker signals for both surface and worm tissue. We therefore further investigated the PE lipid class on a more detailed lipid-species level. Fig 3A shows a diagram depicting the fatty acyl chain lengths versus the number of double bonds in detected PE lipids. Signals found with elevated intensities in the worm tissue are shown as blue cross markers, signals elevated in the surface measurements are shown as orange symbols, and unspecific signals as green squares. Signals classified as both specific and -unspecific (e.g. PE (38:2), see Fig 3A) originated from the presence of multiple *m*/*z*-values caused by different adducts formed. While one adduct was detected as specific, another ion of the same lipid stayed below the threshold of being classified as specific.

The PE composition of surface marker signals appears to be systematically different form tissue-specific signals. Therefore, mean values of fatty acyl chain lengths and number of double bonds were calculated for each class (Fig 3B). Error bars represent the standard deviation between the PEs observed in each class. Herein, differences between surface (in orange) and worm body (in blue) with respect to fatty acyl chain lengths were observed. PE with decreased chain length were predominantly detected on the surface compared to the inner tissue. These trends were preserved when removing putative isobaric interferences of PE/PC as shown in S6 Fig. A decrease in length of the fatty acyl substituents might be associated with decreasing gel-to-liquid-crystalline phase transition (T_M_), a measure for temperature stability of membranes, and therefore the temperature required to induce a disordered liquid crystalline phase, thus disrupting the ordered gel phase.[45] For *S*. *mansoni*, this may indicate that the tegument-associated-surface is more fluidic and thus less rigid compared to the inside. The fluidity could be correlated with the flexibility of the tegument needed for e.g. encapsulating the female during pairing and the subsequent migration of the couple as well as for the high metabolic activity of the tegument. Previously, an LC-MS based study found no differences in PE composition between tegument and worm body, most probably due to lacking methodical accuracy (overlapping error bars, lack of statistical significance).[10] In total, 17 PE species, all of which contain even-numbered fatty acid chains and at least one double bond, were found and quantified in their study.[10] Based on LC coupled with highly sensitive, high-resolution mass spectrometry, we were able to identify 42 PEs (see Fig 3A), some of which contained odd-numbered fatty acid chains (see raw data in S7 Fig) or fully saturated species. To verify that the PE assignments were correctly determined and to rule out isobaric bias, plots of *m*/*z* vs signal intensity were exemplarily assessed as shown in S8 Fig. Statistical categorization and assignment of quasi–isobaric PEs were confirmed for those species discriminable by MS. In total, 20 and 9 signals assigned to PEs were found to specifically occur in worm tissue and the tegument, respectively. PE (39:4) was detected as protonated species and as sodium adduct. The protonated ion was found to be specific for the worm surface, while the sodium adduct was found specifically in the inner worm tissue (see S9 Fig). This rare observation might indicate that either the sodium distribution in the worm was uneven or the ion signal intensities were influenced by an unknown isobaric interference.

Modeling the tegumental membrane of schistosomes could help to understand the complex tegumental surface structure in more detail, as well as lipid conversion processes such as PS synthesis.

### Comparison of tegumental lipid composition of adult male and female *S*. *mansoni*

**Fig 4:**
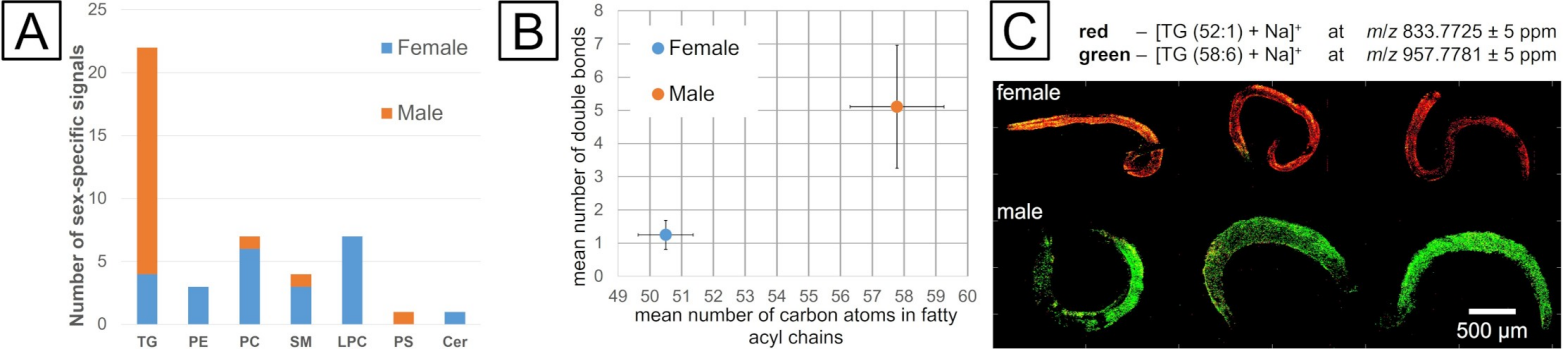
HC of the lipid data set to determine tegumental markers for male and female *S*. *mansoni*. A–Sum composition prior to statistical analysis (input) and classification based on hierarchical clustering into categories of male- and female-specific signals. B–Differences between male and female in triglyceride composition of the tegument with regard to mean number of carbon atoms in fatty acyl chains and number of double bonds. Error bars represent the standard deviation in each biological class. C–Red-Green (RG)-overlay of marker signals [TG (52:1)+Na]^+^ (red) and [TG (58:6)+Na]^+^ (green) for females (top) and males (bottom).

Male and female *S*. *mansoni* worms are morphologically discriminable, as the male contains a gynaecophoric canal and is thicker, due to a higher mass of musculature, but shorter compared to the female. After infection of humans, both sexes occur in identical environments, first the portal vein of the liver and later the mesenteric veins of the gut. The constant pairing contact, however, slightly changes this situation as the proportion of the dorsal surface of the female that is exposed to the host declines, since it is now mainly facing the gynaecophoric canal, the ventral part of the tegument of the male. Previous studies already demonstrated a remarkable consequence of pairing on the transcriptomes and proteomes of schistosome males and females following pairing.[3, 50, 51] Consequently, also differences in the lipid composition on the surface of male and female *S*. *mansoni* worms were hypothesized. Computational analyses were performed on the basis of the same dataset that was used to compare the lipid composition of *S*. *mansoni* surface and sections (Fig 1) to unravel sex-specific tegumental lipid-composition. The sum composition is identical to the aforementioned sum composition in Fig 2. Statistical analysis and HC provided differentially abundant signals for male (Fig 4A, in orange colour) and female (Fig 4A, in blue colour) worms. Additionally, composition of unspecific signals was similar to the sum composition. Differences between male and female surfaces seemed to be less pronounced compared to differences between surface and inner tissue. However, the surface of females was significantly enriched in phospholipids (LPC, PC and PE), sphingolipids (Cer and SM) and TG. A more detailed description of all classified lipids can be found in S4 Table. A former MSI study found TGs only in male but not female schistosomes, probably because worm couples were not separated prior to surface analysis.[21]

Especially molecules which can function in upstream signaling cascades are of high interest to gain insights into male-female interaction. Changes in Cer, SM and LPC are known to be strongly involved in signaling pathways and corresponding upstream signaling.[52, 53] However, more data are required to unravel the function of male- and female-specific surface lipids. For males, TG was the predominantly occurring lipid species, while one signal of each PC, PS and SM per class was more abundant when compared to females.

TG lipid species were found to be specific for both the surface of either males or females. For further investigations, the number of carbon atoms in fatty acyl chains was plotted against the number of double bonds in Fig 4B. The error bars represent the standard deviation of all TG species per class. TGs found in females are marked blue, species that were found in males are marked orange. Signals with increased intensities in females comprised shorter fatty acyl substituents and a decreased number of double bonds compared to the TG species in males. A decrease in fatty acyl chain lengths leads to an increase in fluidity, while the fluidity of TGs is proportional to fatty acyl unsaturation. The degree of unsaturation has a larger impact on tegument fluidity compared to the chain length of fatty acids. In sum this leads to increased fluidity and thus decreased rigidity in TGs on the surface of males. The reason for this difference might be that the males’ dorsal and ventral tegumental surfaces are directed towards the host and the female, respectively. Trans–tegumental nutrient uptake has to be performed by the males’ dorsal tegument, whereas the ventral tegument is involved in male-female inter-tegumental signal transduction processes, because the ventral male tegument covers the tegumental surface of the female. Alternatively, accumulation of TG containing host-derived LDL-particles can occur,[54] and the tegumental surface of the male, available for binding of lipoprotein particles, is larger compared to the female while mated. Investigations of hamster whole blood by extraction and nESI-MS (data not shown) showed partially the same TG lipid species as obtained by MSI (shown in Fig 4). Therefore, TGs found on the surface might be explained by host-derived lipoprotein particles accumulating on the surface of schistosomes, which have been linked to immune evasion in *S*. *mansoni*.[9]

Differential lipid compositions between male and female were also visualized through the MS ion images shown in Fig 4C. The red colour channel shows *m*/*z* 833.7725, which is more abundant in female worms and has been assigned to TG (52:1) as sodiated molecule. The green ion channel represents a signal at *m*/*z* 957.7781 that was assigned to TG (58:6) found as a surface marker for male worms. The visualization of marker lipids for surfaces of male and female *S*. *mansoni* via ion images demonstrated that hierarchical clustering is a valuable statistical tool here, allowing for the reliable determination of signals with significantly altered abundance when comparing two or more sample cohorts.

## Conclusions

AP-SMALDI MSI was used for spatially resolved investigation of lipids in adult *S*. *mansoni* couples, supported by lipid identification using LC-MS/MS for more reliable compound annotation. Authentic signal intensities were obtained from complex non-planar topographies, taking advantage of a novel autofocusing technology. The comparison of worm surface and inner tissues unraveled characteristic differences in composition at the lipid-class level. PC and PE were found to be more abundant inside the worms, while higher abundances of SM, PS, PE and LPC were observed at the surface. The number of carbon atoms in fatty acyl chains and the number of double bonds were investigated in detail, because PE lipid species were characteristic for both surface and inner tissue. Fatty acyl chain lengths of PE were found to be decreased at the surface, which may be associated with decreased rigidity.

Differential lipid compositions of male and female surfaces were also analyzed. Several lipids of TG class were found characteristic for the surface of one of the sexes. Decreased fatty acyl chain lengths in females and higher desaturation in males were detected, hinting towards increased membrane fluidity.

The advantage of AP-SMALDI MSI over classical, LC-MS based lipidomic methods is that the spatial distribution of a wide variety of compounds can be determined immediately from the tissue in a semi-quantitative manner. However, some isobaric lipid species like PEs and PCs are not easily discriminated based on MSI data and have to be identified using fragmentation experiments. At high spatial resolution (here 5 μm), the number of generated ions per spot drastically decreases, and fragmentation experiments are often hardly feasible. This might be overcome by further instrumental improvements. LC-based techniques on the other hand require time-consuming extraction methods, considered to be representative for different tissue types but putatively generating a bias. However, a wide variety of compounds and otherwise isobaric species can be separated, quantified, and identified by fragmentation experiments rapidly and in more detail. Therefore, both techniques (LC-MS/MS and MSI) in combination provide a promising platform for a comprehensive analysis.

For the first time, MSI has been used as a tool to characterize *S*. *mansoni* tegumental surfaces in comparison to whole-worm tissue sections. The suitability of AP-SMALDI MSI was demonstrated to explore locally different lipidomes. Furthermore, this approach allowed to associate specific lipid classes to tissue areas as well as to sex-specific differences at the whole-worm level and thus to testable hypotheses about their potential functions.

## Supporting information

S1 Table Specifications of chemicals used in experimental section. (DOCX)

S2 Table UHPLC-MS method for identification of lipids in S. mansoni. Injection volume was 50 μL. (DOCX)

S3 Table Detailed lipid annotations of classified lipids from comparison of tegument and inner worm tissue. (DOCX)

S4 Table Detailed lipid annotations of classified lipids from comparison of male and female tegument. (DOCX)

S1 Fig Graphical illustration of LC-MS^2^ data analysis workflow. (TIF)

S2 Fig Graphical illustration of the data analysis workflow for MS imaging data. Statistical evaluation work flow was adapted from literature. The statistical analysis comprised five key steps: 1. normalization of one signal to the sum of all signals per measurement, 2. z-score (using median), 3. multiple-class analysis of variance (ANOVA, permutation based false-discovery-rate, FDR, set to 5%, 250 restarts), 4. post-hoc test (5% FDR) and 5. hierarchical clustering (Euclidean distance using average linkage, preprocessing with k-means, maximum 10 iterations, 10 restarts).
(TIF)

S3 Fig Graphical illustration (from Perseus[41]) of multivariate statistical analysis and categorization of differentially abundant signals from MALDI experiments by hierarchical clustering. (TIF)

S4 Fig **Digital light microscopic images of male (M) surfaces (left), female (F) surfaces (middle) and cryosections of couples (right). The black arrows indicate the anterior end**.
(TIF)

S5 Fig Distribution of previously reported most abundant lipid species in *S. mansoni* as protonated and sodiated ion species[20] PC (34:1) has been determined in the past to be differentially abundant in whole worm and tegument. [10] However, MS imaging data did not show significant differences based on HC. For PC (36:1) and PC (36:2), however, our findings are well in accordance with previous publications which found higher abundances inside the worm.[10] The same trend is suggested by unsupervised MS imaging data evaluation presented here.
(TIF)

S6 Fig Number of carbon atoms in fatty acyl chains vs the number of double bonds detected in phosphatidylethanolamines (PE). Isobaric PE/PC interferences were excluded for surface and section data. A–Comparison of worm-tissue (blue cross) and surface/tegument specific signals vs ions (orange +) with unspecific distribution (green square). Overlapping indicators are attributed to the presence of several adducts corresponding to one lipid species. B–Arithmetic mean fatty acyl and double bond composition for section/inner tissue (blue), surface/tegument (orange) and unspecific signals (green). Error bars show the standard deviation across one location.
(TIF)

S7 Fig Example for LC-MS/MS based identification of PE (37:4) with MS1 overview spectra and data-dependent MS2 spectra. A–MS1 overview spectrum. B–virtual magnification of mass range m/z 700–800 (from A) showing the mass of PE (37:4) as deprotonated species (C_42_H_75_NO_8_P). C–MS2 spectrum of precursor m/z 752.52 ±0.5 u showing characteristic fragments of PE head group (around m/z 140 and m/z 196), FA (17:0) and FA (20:4). The precursor is not visible in the spectrum and assumedly fragmented quantitatively at NCE = 30. D–virtual magnification of m/z 750–755 (from A) showing mass and isotope ratio of PE 37:4 as ^12^C, ^13^C and ^13^C_2_ isotopologues.
(TIF)

S8 Fig Example for mass accuracy and resolution obtained in MSI experiments. A–MS ion image of nearly isobaric PE-adduct species [PE (39:5) + H]^+^ and [PE (37:2) + Na]^+^ (Δm = 3.1 ppm) at m/z 780.5537 ± 5 ppm. B–Signal intensity (abundance in NL; normalized level) vs mass deviation in ppm. A double peak can be observed shifted by approximately 1.5 ppm and 2.8 ppm. C–MS ion signal at m/z 780.55254 ± 0.2 ppm showing an increased signal intensity on the worm surface assigned to protonated PE (39:5). D–MS ion at m/z 780.55151 ± 0.2 ppm assigned to PE (37:2) as sodium adduct. By hierarchical clustering, the signal at m/z 780.5537 was determined to be more abundant in the worm body compared to tegumental surface (see Fig 3). The signal was assigned to PE (37:2) as sodiated molecule. The protonated species of PE (39:5), however, was classified as unspecific. The fluctuating signal intensity of the surface measurements putatively led to unspecific classification. This example thus verifies the accuracy and correctness of HC-based classification.
(TIF)

S9 Fig Putative adducts of PE (39:4) with different distributions. A—Distribution of m/z 782.5694 assigned to [PE (39:4) + H]^+^. B–distribution of m/z 804.5514 assigned to [PE (39:4) + Na]^+^. This difference in distribution could be explained by different concentrations of salt in tegument and inner tissue or by isobaric interferences that were not contained in the LC-MS/MS-database.
(TIF)
