# Supplementary material for: Tissue- and sex-specific lipidomic analysis of Schistosoma mansoni using high-resolution atmospheric pressure scanning microprobe matrix-assisted laser desorption/ionization mass spectrometry imaging
Source: PLoS Negl Trop Dis. 2020 May 13;14(5):e0008145. doi: 10.1371/journal.pntd.0008145 (PMC7250470; doi:10.1371/journal.pntd.0008145)
Supplement: S1 Table — (DOCX) [file pntd.0008145.s001.docx]

| Chemical name | Quality grade | Manufacturer |
| --- | --- | --- |
| glutaraldehyde | grade I | Sigma-Aldrich, St. Louis, MO, USA |
| phosphate buffered saline | Gibco | Thermo Fisher Scientific, Bremen, Germany |
| gelatin | pharm. Eur. | VWR, Radnor, PA, USA |
| Methyl-tert butylether | for HPLC | Sigma-Aldrich, St. Louis, MO, USA |
| methanol | LiChroSolv | Merck, Darmstadt, Germany |
| water | LC-MS grade | VWR, Radnor, PA, USA |
| formic acid | for mass spectrometry | Honeywell, Morris Plains, NJ, USA |
| ammonium formate | 99.995% | Sigma-Aldrich, St. Louis, MO, USA |
| 2-propanol | for HPLC | VWR, Radnor, PA, USA |
| ammonium acetate | LC-MS grade | Honeywell, Morris Plains, NJ, USA |
| 2,5-dihydroxy benzoic acid | for synthesis | Merck, Darmstadt, Germany |
| acetone | Uvasol | Merck, Darmstadt, Germany |
| trifluoro acetic acid | for spectroscopy | AppliChem, Darmstadt, Germany |
