# Supplementary material for: Tissue- and sex-specific lipidomic analysis of Schistosoma mansoni using high-resolution atmospheric pressure scanning microprobe matrix-assisted laser desorption/ionization mass spectrometry imaging
Source: PLoS Negl Trop Dis. 2020 May 13;14(5):e0008145. doi: 10.1371/journal.pntd.0008145 (PMC7250470; doi:10.1371/journal.pntd.0008145)
Supplement: S2 Table — (DOCX) [file pntd.0008145.s002.docx]

| UHPLC-method | | | MS-method | |
| --- | --- | --- | --- | --- |
| flow rate in µL/min | | 90 | **MS^1^** | |
| V(injection) in µL | | 50 |  |  |
| T(autosampler) in °C | | 5 | *m*/*z*-range | ± 300-1600 |
| T(column oven) in °C | | 50 | automatic gain control | 10^6^ |
| stationary phase | | BEH C18 Acquity (Waters, Milford, MA, USA), 150 x 1 mm, 1.7 µm particle size | Lock-masses (positive polarity only) | *m*/*z* 391.2843 and *m*/*z* 610.1842[55] |
| mobile phase A | | water:methanol:2-propanol 6:5:9 v/v/v, 0.1% formic acid and 10 mM ammonium formate | **MS^2^** | |
| mobile phase B | | methanol:2-propanol 1:9 v/v, 0.1% formic acid and 10 mM ammonium formate | fragmentation mechanism | C-trap collisional dissociation (HCD) |
| t in min | %B | | fragmentation energy in normalized collision energy (NCE) | 25 (pos.) 30 (neg.) |
| 0 | | 0 | *m*/*z*-selection | ten most intense |
| 1 | | 0 | automatic gain control | 10^5^ |
| 31 | | 100 | isolation width | ± 0.5 Da |
| 41 | | 100 | charge inclusion | 1 and 2 |
| 43 | | 0 | isotope exclusion | active |
| 50 | | 0 | dynamic exclusion in s | 15 |
