# Supplementary material for: Tissue- and sex-specific lipidomic analysis of Schistosoma mansoni using high-resolution atmospheric pressure scanning microprobe matrix-assisted laser desorption/ionization mass spectrometry imaging
Source: PLoS Negl Trop Dis. 2020 May 13;14(5):e0008145. doi: 10.1371/journal.pntd.0008145 (PMC7250470; doi:10.1371/journal.pntd.0008145)
Supplement: S4 Table — (DOCX) [file pntd.0008145.s004.docx]

|  |  |  |  |  | **According to SwissLipids database** | | | |  |
| --- | --- | --- | --- | --- | --- | --- | --- | --- | --- |
| **Location with increased signal intenisty** | **Abbreviation** | **Adduct** | **formula** | **mz** | **Lipid ID** | **Lipid class** | **SMILES (pH7.3)** | **CHEBI** | **Number of MSI datasets with positive annotation by Metaspace** |
| Female | TG(52:1) | M+Na | C55H104O6 | 883,7724902 | SLM:000308296 | SLM:000000400 | [*]C(=O)OCC(COC([*])=O)OC([*])=O | 0 | 5 |
| Female | PC(40:6) | M+H | C48H84NO8P | 834,6007096 | SLM:000056561 | SLM:000055211 | C[N+](C)(C)CCOP([O-])(=O)OCC(COC([*])=O)OC([*])=O | 64431 | 4 |
| Female | PE(41:3) | M+Na | C46H86NO8P | 834,5983043 | SLM:000057226 | SLM:000055213 | [NH3+]CCOP([O-])(=O)OCC(COC([*])=O)OC([*])=O | 0 | 5 |
| Female | SM(d36:1) | M+K | C41H83N2O6P | 769,5620112 | SLM:000390739 | SLM:000001000 | C[N+](C)(C)CCOP([O-])(=O)OC[C@H](NC([*])=O)[C@H](O)[*] | 0 | 9 |
| Female | PC(42:9) | M+H | C50H82NO8P | 856,5850596 | SLM:000056583 | SLM:000055211 | C[N+](C)(C)CCOP([O-])(=O)OCC(COC([*])=O)OC([*])=O | 0 | 9 |
| Female | PC(40:6) | M+Na | C48H84NO8P | 856,5826543 | SLM:000056561 | SLM:000055211 | C[N+](C)(C)CCOP([O-])(=O)OCC(COC([*])=O)OC([*])=O | 64431 | 4 |
| Female | TG(50:1) | M+K | C53H100O6 | 871,7151273 | SLM:000308276 | SLM:000000400 | [*]C(=O)OCC(COC([*])=O)OC([*])=O | 0 | 5 |
| Female | SM(d43:1) | M+H | C48H97N2O6P | 829,7156798 | SLM:000390838 | SLM:000001000 | C[N+](C)(C)CCOP([O-])(=O)OC[C@H](NC([*])=O)[C@H](O)[*] | 0 | 3 |
| Female | PC(40:7) | M+H | C48H82NO8P | 832,5850596 | SLM:000056562 | SLM:000055211 | C[N+](C)(C)CCOP([O-])(=O)OCC(COC([*])=O)OC([*])=O | 0 | 9 |
| Female | PE(41:4) | M+Na | C46H84NO8P | 832,5826543 | SLM:000057227 | SLM:000055213 | [NH3+]CCOP([O-])(=O)OCC(COC([*])=O)OC([*])=O | 0 | 8 |
| Female | LPC(20:1) | M+H | C28H56NO7P | 550,3866941 | SLM:000055329 | SLM:000055200 | C[N+](C)(C)CCOP([O-])(=O)OCC(CO[*])O[*] | 67057 | 9 |
| Female | PC(42:7) | M+H | C50H86NO8P | 860,6163597 | SLM:000056581 | SLM:000055211 | C[N+](C)(C)CCOP([O-])(=O)OCC(COC([*])=O)OC([*])=O | 0 | 6 |
| Female | PE(43:4) | M+Na | C48H88NO8P | 860,6139544 | SLM:000057246 | SLM:000055213 | [NH3+]CCOP([O-])(=O)OCC(COC([*])=O)OC([*])=O | 0 | 5 |
| Female | TG(50:1) | M+Na | C53H100O6 | 855,74119 | SLM:000308276 | SLM:000000400 | [*]C(=O)OCC(COC([*])=O)OC([*])=O | 0 | 5 |
| Female | TG(50:2) | M+Na | C53H98O6 | 853,72554 | SLM:000308277 | SLM:000000400 | [*]C(=O)OCC(COC([*])=O)OC([*])=O | 0 | 6 |
| Female | LPC(20:2) | M+H | C28H54NO7P | 548,3710441 | SLM:000055330 | SLM:000055200 | C[N+](C)(C)CCOP([O-])(=O)OCC(CO[*])O[*] | 0 | 9 |
| Female | PC(36:0) | M+H | C44H88NO8P | 790,6320098 | SLM:000056521 | SLM:000055211 | C[N+](C)(C)CCOP([O-])(=O)OCC(COC([*])=O)OC([*])=O | 66858 | 9 |
| Female | LPC(22:4) | M+H | C30H54NO7P | 572,3710441 | SLM:000055339 | SLM:000055200 | C[N+](C)(C)CCOP([O-])(=O)OCC(CO[*])O[*] | 0 | 6 |
| Female | LPC(20:1) | M+Na | C28H56NO7P | 572,3686387 | SLM:000055329 | SLM:000055200 | C[N+](C)(C)CCOP([O-])(=O)OCC(CO[*])O[*] | 67057 | 9 |
| Female | LPC(18:2) | M+Na | C26H50NO7P | 542,3216886 | SLM:000055324 | SLM:000055200 | C[N+](C)(C)CCOP([O-])(=O)OCC(CO[*])O[*] | 0 | 9 |
| Female | SM(d43:1) | M+Na | C48H97N2O6P | 851,6976244 | SLM:000390838 | SLM:000001000 | C[N+](C)(C)CCOP([O-])(=O)OC[C@H](NC([*])=O)[C@H](O)[*] | 0 | 3 |
| Female | LPC(22:5) | M+H | C30H52NO7P | 570,355394 | SLM:000055340 | SLM:000055200 | C[N+](C)(C)CCOP([O-])(=O)OCC(CO[*])O[*] | 0 | 8 |
| Female | LPC(20:2) | M+Na | C28H54NO7P | 570,3529887 | SLM:000055330 | SLM:000055200 | C[N+](C)(C)CCOP([O-])(=O)OCC(CO[*])O[*] | 0 | 9 |
| Female | Cer(d34:1) | M+H | C34H67NO3 | 538,5193495 | SLM:000391236 | SLM:000399814 | OC[C@H](NC([*])=O)[C@H](O)[*] | 0 | 3 |
| Male | TG(60:7) | M+H | C63H108O6 | 961,8218457 | SLM:000308410 | SLM:000000400 | [*]C(=O)OCC(COC([*])=O)OC([*])=O | 0 | 3 |
| Male | TG(58:4) | M+Na | C61H110O6 | 961,8194404 | SLM:000308378 | SLM:000000400 | [*]C(=O)OCC(COC([*])=O)OC([*])=O | 0 | 6 |
| Male | TG(58:7) | M+H | C61H104O6 | 933,7905455 | SLM:000308381 | SLM:000000400 | [*]C(=O)OCC(COC([*])=O)OC([*])=O | 0 | 5 |
| Male | TG(56:4) | M+Na | C59H106O6 | 933,7881402 | SLM:000308350 | SLM:000000400 | [*]C(=O)OCC(COC([*])=O)OC([*])=O | 0 | 6 |
| Male | TG(58:5) | M+H | C61H108O6 | 937,8218457 | SLM:000308379 | SLM:000000400 | [*]C(=O)OCC(COC([*])=O)OC([*])=O | 0 | 5 |
| Male | TG(56:2) | M+Na | C59H110O6 | 937,8194404 | SLM:000308348 | SLM:000000400 | [*]C(=O)OCC(COC([*])=O)OC([*])=O | 0 | 8 |
| Male | TG(58:3) | M+Na | C61H112O6 | 963,8350904 | SLM:000308377 | SLM:000000400 | [*]C(=O)OCC(COC([*])=O)OC([*])=O | 0 | 8 |
| Male | TG(60:6) | M+H | C63H110O6 | 963,8374957 | SLM:000308409 | SLM:000000400 | [*]C(=O)OCC(COC([*])=O)OC([*])=O | 0 | 8 |
| Male | TG(58:6) | M+H | C61H106O6 | 935,8061956 | SLM:000308380 | SLM:000000400 | [*]C(=O)OCC(COC([*])=O)OC([*])=O | 0 | 5 |
| Male | TG(56:3) | M+Na | C59H108O6 | 935,8037903 | SLM:000308349 | SLM:000000400 | [*]C(=O)OCC(COC([*])=O)OC([*])=O | 0 | 6 |
| Male | TG(60:8) | M+H | C63H106O6 | 959,8061956 | SLM:000308411 | SLM:000000400 | [*]C(=O)OCC(COC([*])=O)OC([*])=O | 0 | 8 |
| Male | TG(58:5) | M+Na | C61H108O6 | 959,8037903 | SLM:000308379 | SLM:000000400 | [*]C(=O)OCC(COC([*])=O)OC([*])=O | 0 | 5 |
| Male | SM(d38:2) | M+Na | C43H85N2O6P | 779,6037241 | SLM:000390765 | SLM:000001000 | C[N+](C)(C)CCOP([O-])(=O)OC[C@H](NC([*])=O)[C@H](O)[*] | 0 | 7 |
| Male | TG(60:9) | M+H | C63H104O6 | 957,7905455 | SLM:000308412 | SLM:000000400 | [*]C(=O)OCC(COC([*])=O)OC([*])=O | 0 | 8 |
| Male | TG(58:6) | M+Na | C61H106O6 | 957,7881402 | SLM:000308380 | SLM:000000400 | [*]C(=O)OCC(COC([*])=O)OC([*])=O | 0 | 5 |
| Male | PS(38:1) | M+K | C44H84NO10P | 856,5464207 | SLM:000058892 | SLM:000055218 | [NH3+][C@@H](COP([O-])(=O)OCC(COC([*])=O)OC([*])=O)C([O-])=O | 72072 | 8 |
| Male | PC(30:0) | M+H | C38H76NO8P | 706,5381094 | SLM:000056479 | SLM:000055211 | C[N+](C)(C)CCOP([O-])(=O)OCC(COC([*])=O)OC([*])=O | 65303 | 9 |
| Male | TG(56:4) | M+K | C59H106O6 | 949,7620775 | SLM:000308350 | SLM:000000400 | [*]C(=O)OCC(COC([*])=O)OC([*])=O | 0 | 6 |
| Male | TG(56:6) | M+H | C59H102O6 | 907,7748955 | SLM:000308352 | SLM:000000400 | [*]C(=O)OCC(COC([*])=O)OC([*])=O | 0 | 6 |
| Male | TG(56:3) | M+K | C59H108O6 | 951,7777275 | SLM:000308349 | SLM:000000400 | [*]C(=O)OCC(COC([*])=O)OC([*])=O | 0 | 6 |
| Male | TG(58:4) | M+K | C61H110O6 | 977,7933776 | SLM:000308378 | SLM:000000400 | [*]C(=O)OCC(COC([*])=O)OC([*])=O | 0 | 6 |
| Unspecific | TG(64:8) | M+H | C67H114O6 | 1015,868796 | SLM:000308472 | SLM:000000400 | [*]C(=O)OCC(COC([*])=O)OC([*])=O | 0 | 4 |
| Unspecific | TG(62:5) | M+Na | C65H116O6 | 1015,866391 | SLM:000308438 | SLM:000000400 | [*]C(=O)OCC(COC([*])=O)OC([*])=O | 0 | 3 |
| Unspecific | TG(60:7) | M+K | C63H108O6 | 999,7777275 | SLM:000308410 | SLM:000000400 | [*]C(=O)OCC(COC([*])=O)OC([*])=O | 0 | 3 |
| Unspecific | TG(62:7) | M+H | C65H112O6 | 989,8531458 | SLM:000308440 | SLM:000000400 | [*]C(=O)OCC(COC([*])=O)OC([*])=O | 0 | 4 |
| Unspecific | TG(60:4) | M+Na | C63H114O6 | 989,8507405 | SLM:000308407 | SLM:000000400 | [*]C(=O)OCC(COC([*])=O)OC([*])=O | 0 | 7 |
| Unspecific | TG(62:8) | M+H | C65H110O6 | 987,8374957 | SLM:000308441 | SLM:000000400 | [*]C(=O)OCC(COC([*])=O)OC([*])=O | 0 | 4 |
| Unspecific | TG(60:5) | M+Na | C63H112O6 | 987,8350904 | SLM:000308408 | SLM:000000400 | [*]C(=O)OCC(COC([*])=O)OC([*])=O | 0 | 7 |
| Unspecific | TG(62:9) | M+H | C65H108O6 | 985,8218457 | SLM:000308442 | SLM:000000400 | [*]C(=O)OCC(COC([*])=O)OC([*])=O | 0 | 4 |
| Unspecific | TG(60:6) | M+Na | C63H110O6 | 985,8194404 | SLM:000308409 | SLM:000000400 | [*]C(=O)OCC(COC([*])=O)OC([*])=O | 0 | 8 |
| Unspecific | TG(62:10) | M+H | C65H106O6 | 983,8061956 | SLM:000308428 | SLM:000000400 | [*]C(=O)OCC(COC([*])=O)OC([*])=O | 0 | 4 |
| Unspecific | TG(60:7) | M+Na | C63H108O6 | 983,8037903 | SLM:000308410 | SLM:000000400 | [*]C(=O)OCC(COC([*])=O)OC([*])=O | 0 | 3 |
| Unspecific | TG(62:11) | M+H | C65H104O6 | 981,7905455 | SLM:000308429 | SLM:000000400 | [*]C(=O)OCC(COC([*])=O)OC([*])=O | 0 | 8 |
| Unspecific | TG(60:8) | M+Na | C63H106O6 | 981,7881402 | SLM:000308411 | SLM:000000400 | [*]C(=O)OCC(COC([*])=O)OC([*])=O | 0 | 8 |
| Unspecific | TG(62:12) | M+H | C65H102O6 | 979,7748955 | SLM:000308430 | SLM:000000400 | [*]C(=O)OCC(COC([*])=O)OC([*])=O | 0 | 7 |
| Unspecific | TG(60:9) | M+Na | C63H104O6 | 979,7724902 | SLM:000308412 | SLM:000000400 | [*]C(=O)OCC(COC([*])=O)OC([*])=O | 0 | 8 |
| Unspecific | TG(58:10) | M+K | C61H98O6 | 965,6994772 | SLM:000308371 | SLM:000000400 | [*]C(=O)OCC(COC([*])=O)OC([*])=O | 0 | 3 |
| Unspecific | TG(60:10) | M+H | C63H102O6 | 955,7748955 | SLM:000308399 | SLM:000000400 | [*]C(=O)OCC(COC([*])=O)OC([*])=O | 0 | 6 |
| Unspecific | TG(58:7) | M+Na | C61H104O6 | 955,7724902 | SLM:000308381 | SLM:000000400 | [*]C(=O)OCC(COC([*])=O)OC([*])=O | 0 | 5 |
| Unspecific | TG(60:11) | M+H | C63H100O6 | 953,7592454 | SLM:000308400 | SLM:000000400 | [*]C(=O)OCC(COC([*])=O)OC([*])=O | 0 | 9 |
| Unspecific | TG(58:8) | M+Na | C61H102O6 | 953,7568401 | SLM:000308382 | SLM:000000400 | [*]C(=O)OCC(COC([*])=O)OC([*])=O | 0 | 3 |
| Unspecific | TG(60:12) | M+H | C63H98O6 | 951,7435953 | SLM:000308401 | SLM:000000400 | [*]C(=O)OCC(COC([*])=O)OC([*])=O | 0 | 7 |
| Unspecific | TG(58:9) | M+Na | C61H100O6 | 951,74119 | SLM:000308383 | SLM:000000400 | [*]C(=O)OCC(COC([*])=O)OC([*])=O | 0 | 8 |
| Unspecific | TG(58:10) | M+Na | C61H98O6 | 949,72554 | SLM:000308371 | SLM:000000400 | [*]C(=O)OCC(COC([*])=O)OC([*])=O | 0 | 3 |
| Unspecific | TG(56:5) | M+K | C59H104O6 | 947,7464274 | SLM:000308351 | SLM:000000400 | [*]C(=O)OCC(COC([*])=O)OC([*])=O | 0 | 6 |
| Unspecific | TG(57:5) | M+Na | C60H106O6 | 945,7881402 | SLM:000308364 | SLM:000000400 | [*]C(=O)OCC(COC([*])=O)OC([*])=O | 0 | 3 |
| Unspecific | TG(56:6) | M+K | C59H102O6 | 945,7307773 | SLM:000308352 | SLM:000000400 | [*]C(=O)OCC(COC([*])=O)OC([*])=O | 0 | 6 |
| Unspecific | TG(57:6) | M+Na | C60H104O6 | 943,7724902 | SLM:000308365 | SLM:000000400 | [*]C(=O)OCC(COC([*])=O)OC([*])=O | 0 | 3 |
| Unspecific | TG(56:8) | M+K | C59H98O6 | 941,6994772 | SLM:000308354 | SLM:000000400 | [*]C(=O)OCC(COC([*])=O)OC([*])=O | 0 | 3 |
| Unspecific | TG(58:4) | M+H | C61H110O6 | 939,8374957 | SLM:000308378 | SLM:000000400 | [*]C(=O)OCC(COC([*])=O)OC([*])=O | 0 | 6 |
| Unspecific | TG(58:8) | M+H | C61H102O6 | 931,7748955 | SLM:000308382 | SLM:000000400 | [*]C(=O)OCC(COC([*])=O)OC([*])=O | 0 | 3 |
| Unspecific | TG(56:5) | M+Na | C59H104O6 | 931,7724902 | SLM:000308351 | SLM:000000400 | [*]C(=O)OCC(COC([*])=O)OC([*])=O | 0 | 6 |
| Unspecific | TG(58:9) | M+H | C61H100O6 | 929,7592454 | SLM:000308383 | SLM:000000400 | [*]C(=O)OCC(COC([*])=O)OC([*])=O | 0 | 8 |
| Unspecific | TG(56:6) | M+Na | C59H102O6 | 929,7568401 | SLM:000308352 | SLM:000000400 | [*]C(=O)OCC(COC([*])=O)OC([*])=O | 0 | 6 |
| Unspecific | TG(58:10) | M+H | C61H98O6 | 927,7435953 | SLM:000308371 | SLM:000000400 | [*]C(=O)OCC(COC([*])=O)OC([*])=O | 0 | 3 |
| Unspecific | TG(56:7) | M+Na | C59H100O6 | 927,74119 | SLM:000308353 | SLM:000000400 | [*]C(=O)OCC(COC([*])=O)OC([*])=O | 0 | 5 |
| Unspecific | TG(54:2) | M+K | C57H106O6 | 925,7620775 | SLM:000308322 | SLM:000000400 | [*]C(=O)OCC(COC([*])=O)OC([*])=O | 0 | 6 |
| Unspecific | TG(58:11) | M+H | C61H96O6 | 925,7279453 | SLM:000308372 | SLM:000000400 | [*]C(=O)OCC(COC([*])=O)OC([*])=O | 0 | 8 |
| Unspecific | TG(56:8) | M+Na | C59H98O6 | 925,72554 | SLM:000308354 | SLM:000000400 | [*]C(=O)OCC(COC([*])=O)OC([*])=O | 0 | 3 |
| Unspecific | PI(38:4) | M+K | C47H83O13P | 925,5202656 | SLM:000058237 | SLM:000055216 | OC1C(O)C(O)C(OP([O-])(=O)OCC(COC([*])=O)OC([*])=O)C(O)C1O | 0 | 3 |
| Unspecific | TG(56:9) | M+Na | C59H96O6 | 923,7098899 | SLM:000308355 | SLM:000000400 | [*]C(=O)OCC(COC([*])=O)OC([*])=O | 0 | 3 |
| Unspecific | TG(54:5) | M+K | C57H100O6 | 919,7151273 | SLM:000308325 | SLM:000000400 | [*]C(=O)OCC(COC([*])=O)OC([*])=O | 0 | 5 |
| Unspecific | TG(56:3) | M+H | C59H108O6 | 913,8218457 | SLM:000308349 | SLM:000000400 | [*]C(=O)OCC(COC([*])=O)OC([*])=O | 0 | 6 |
| Unspecific | PS(42:1) | M+K | C48H92NO10P | 912,609021 | SLM:000058927 | SLM:000055218 | [NH3+][C@@H](COP([O-])(=O)OCC(COC([*])=O)OC([*])=O)C([O-])=O | 72086 | 9 |
| Unspecific | PS(42:2) | M+K | C48H90NO10P | 910,5933709 | SLM:000058930 | SLM:000055218 | [NH3+][C@@H](COP([O-])(=O)OCC(COC([*])=O)OC([*])=O)C([O-])=O | 72087 | 5 |
| Unspecific | TG(56:5) | M+H | C59H104O6 | 909,7905455 | SLM:000308351 | SLM:000000400 | [*]C(=O)OCC(COC([*])=O)OC([*])=O | 0 | 6 |
| Unspecific | TG(54:2) | M+Na | C57H106O6 | 909,7881402 | SLM:000308322 | SLM:000000400 | [*]C(=O)OCC(COC([*])=O)OC([*])=O | 0 | 6 |
| Unspecific | PI(40:7) | M+H | C49H81O13P | 909,5487336 | SLM:000058258 | SLM:000055216 | OC1C(O)C(O)C(OP([O-])(=O)OCC(COC([*])=O)OC([*])=O)C(O)C1O | 0 | 4 |
| Unspecific | PI(38:5) | M+Na | C47H81O13P | 907,5306783 | SLM:000058238 | SLM:000055216 | OC1C(O)C(O)C(OP([O-])(=O)OCC(COC([*])=O)OC([*])=O)C(O)C1O | 0 | 4 |
| Unspecific | TG(56:7) | M+H | C59H100O6 | 905,7592454 | SLM:000308353 | SLM:000000400 | [*]C(=O)OCC(COC([*])=O)OC([*])=O | 0 | 5 |
| Unspecific | PS(43:4) | M+Na | C49H88NO10P | 904,6037836 | SLM:000058942 | SLM:000055218 | [NH3+][C@@H](COP([O-])(=O)OCC(COC([*])=O)OC([*])=O)C([O-])=O | 0 | 9 |
| Unspecific | TG(56:8) | M+H | C59H98O6 | 903,7435953 | SLM:000308354 | SLM:000000400 | [*]C(=O)OCC(COC([*])=O)OC([*])=O | 0 | 3 |
| Unspecific | TG(54:5) | M+Na | C57H100O6 | 903,74119 | SLM:000308325 | SLM:000000400 | [*]C(=O)OCC(COC([*])=O)OC([*])=O | 0 | 5 |
| Unspecific | TG(56:9) | M+H | C59H96O6 | 901,7279453 | SLM:000308355 | SLM:000000400 | [*]C(=O)OCC(COC([*])=O)OC([*])=O | 0 | 3 |
| Unspecific | TG(52:1) | M+K | C55H104O6 | 899,7464274 | SLM:000308296 | SLM:000000400 | [*]C(=O)OCC(COC([*])=O)OC([*])=O | 0 | 5 |
| Unspecific | PC(42:1) | M+Na | C50H98NO8P | 894,6922047 | SLM:000056573 | SLM:000055211 | C[N+](C)(C)CCOP([O-])(=O)OCC(COC([*])=O)OC([*])=O | 66970 | 4 |
| Unspecific | PC(44:5) | M+H | C52H94NO8P | 892,67896 | SLM:000056599 | SLM:000055211 | C[N+](C)(C)CCOP([O-])(=O)OCC(COC([*])=O)OC([*])=O | 66974 | 3 |
| Unspecific | PC(42:2) | M+Na | C50H96NO8P | 892,6765546 | SLM:000056576 | SLM:000055211 | C[N+](C)(C)CCOP([O-])(=O)OCC(COC([*])=O)OC([*])=O | 66969 | 3 |
| Unspecific | PC(44:6) | M+H | C52H92NO8P | 890,6633099 | SLM:000056600 | SLM:000055211 | C[N+](C)(C)CCOP([O-])(=O)OCC(COC([*])=O)OC([*])=O | 66973 | 3 |
| Unspecific | PC(42:3) | M+Na | C50H94NO8P | 890,6609046 | SLM:000056577 | SLM:000055211 | C[N+](C)(C)CCOP([O-])(=O)OCC(COC([*])=O)OC([*])=O | 66968 | 3 |
| Unspecific | PI(38:4) | M+H | C47H83O13P | 887,5643837 | SLM:000058237 | SLM:000055216 | OC1C(O)C(O)C(OP([O-])(=O)OCC(COC([*])=O)OC([*])=O)C(O)C1O | 0 | 3 |
| Unspecific | PI(36:1) | M+Na | C45H85O13P | 887,5619784 | SLM:000058218 | SLM:000055216 | OC1C(O)C(O)C(OP([O-])(=O)OCC(COC([*])=O)OC([*])=O)C(O)C1O | 74371 | 9 |
| Unspecific | PC(44:8) | M+H | C52H88NO8P | 886,6320098 | SLM:000056602 | SLM:000055211 | C[N+](C)(C)CCOP([O-])(=O)OCC(COC([*])=O)OC([*])=O | 0 | 4 |
| Unspecific | PC(42:5) | M+Na | C50H90NO8P | 886,6296045 | SLM:000056579 | SLM:000055211 | C[N+](C)(C)CCOP([O-])(=O)OCC(COC([*])=O)OC([*])=O | 66966 | 7 |
| Unspecific | TG(52:0) | M+Na | C55H106O6 | 885,7881402 | SLM:000308295 | SLM:000000400 | [*]C(=O)OCC(COC([*])=O)OC([*])=O | 0 | 3 |
| Unspecific | PI(38:5) | M+H | C47H81O13P | 885,5487336 | SLM:000058238 | SLM:000055216 | OC1C(O)C(O)C(OP([O-])(=O)OCC(COC([*])=O)OC([*])=O)C(O)C1O | 0 | 4 |
| Unspecific | PI(36:2) | M+Na | C45H83O13P | 885,5463283 | SLM:000058219 | SLM:000055216 | OC1C(O)C(O)C(OP([O-])(=O)OCC(COC([*])=O)OC([*])=O)C(O)C1O | 74372 | 7 |
| Unspecific | PC(42:6) | M+Na | C50H88NO8P | 884,6139544 | SLM:000056580 | SLM:000055211 | C[N+](C)(C)CCOP([O-])(=O)OCC(COC([*])=O)OC([*])=O | 66965 | 7 |
| Unspecific | PS(40:1) | M+K | C46H88NO10P | 884,5777209 | SLM:000058909 | SLM:000055218 | [NH3+][C@@H](COP([O-])(=O)OCC(COC([*])=O)OC([*])=O)C([O-])=O | 72079 | 9 |
| Unspecific | PI(36:3) | M+Na | C45H81O13P | 883,5306783 | SLM:000058220 | SLM:000055216 | OC1C(O)C(O)C(OP([O-])(=O)OCC(COC([*])=O)OC([*])=O)C(O)C1O | 0 | 3 |
| Unspecific | PS(40:2) | M+K | C46H86NO10P | 882,5620708 | SLM:000058911 | SLM:000055218 | [NH3+][C@@H](COP([O-])(=O)OCC(COC([*])=O)OC([*])=O)C([O-])=O | 72080 | 9 |
| Unspecific | TG(54:5) | M+H | C57H100O6 | 881,7592454 | SLM:000308325 | SLM:000000400 | [*]C(=O)OCC(COC([*])=O)OC([*])=O | 0 | 5 |
| Unspecific | TG(52:2) | M+Na | C55H102O6 | 881,7568401 | SLM:000308298 | SLM:000000400 | [*]C(=O)OCC(COC([*])=O)OC([*])=O | 0 | 8 |
| Unspecific | TG(52:3) | M+Na | C55H100O6 | 879,74119 | SLM:000308299 | SLM:000000400 | [*]C(=O)OCC(COC([*])=O)OC([*])=O | 0 | 8 |
| Unspecific | PS(41:3) | M+Na | C47H86NO10P | 878,5881336 | SLM:000058922 | SLM:000055218 | [NH3+][C@@H](COP([O-])(=O)OCC(COC([*])=O)OC([*])=O)C([O-])=O | 0 | 9 |
| Unspecific | PE(44:4) | M+Na | C49H90NO8P | 874,6296045 | SLM:000057256 | SLM:000055213 | [NH3+]CCOP([O-])(=O)OCC(COC([*])=O)OC([*])=O | 71758 | 3 |
| Unspecific | PS(41:5) | M+Na | C47H82NO10P | 874,5568334 | SLM:000058924 | SLM:000055218 | [NH3+][C@@H](COP([O-])(=O)OCC(COC([*])=O)OC([*])=O)C([O-])=O | 0 | 3 |
| Unspecific | PC(40:6) | M+K | C48H84NO8P | 872,5565915 | SLM:000056561 | SLM:000055211 | C[N+](C)(C)CCOP([O-])(=O)OCC(COC([*])=O)OC([*])=O | 64431 | 4 |
| Unspecific | PS(42:3) | M+H | C48H88NO10P | 870,621839 | SLM:000058931 | SLM:000055218 | [NH3+][C@@H](COP([O-])(=O)OCC(COC([*])=O)OC([*])=O)C([O-])=O | 72088 | 3 |
| Unspecific | TG(53:4) | M+H | C56H100O6 | 869,7592454 | SLM:000308311 | SLM:000000400 | [*]C(=O)OCC(COC([*])=O)OC([*])=O | 0 | 4 |
| Unspecific | TG(51:1) | M+Na | C54H102O6 | 869,7568401 | SLM:000308286 | SLM:000000400 | [*]C(=O)OCC(COC([*])=O)OC([*])=O | 0 | 4 |
| Unspecific | PC(42:3) | M+H | C50H94NO8P | 868,67896 | SLM:000056577 | SLM:000055211 | C[N+](C)(C)CCOP([O-])(=O)OCC(COC([*])=O)OC([*])=O | 66968 | 3 |
| Unspecific | PC(40:0) | M+Na | C48H96NO8P | 868,6765546 | SLM:000056554 | SLM:000055211 | C[N+](C)(C)CCOP([O-])(=O)OCC(COC([*])=O)OC([*])=O | 66866 | 6 |
| Unspecific | PS(42:4) | M+H | C48H86NO10P | 868,6061889 | SLM:000058932 | SLM:000055218 | [NH3+][C@@H](COP([O-])(=O)OCC(COC([*])=O)OC([*])=O)C([O-])=O | 72089 | 3 |
| Unspecific | PS(40:1) | M+Na | C46H88NO10P | 868,6037836 | SLM:000058909 | SLM:000055218 | [NH3+][C@@H](COP([O-])(=O)OCC(COC([*])=O)OC([*])=O)C([O-])=O | 72079 | 9 |
| Unspecific | PC(42:4) | M+H | C50H92NO8P | 866,6633099 | SLM:000056578 | SLM:000055211 | C[N+](C)(C)CCOP([O-])(=O)OCC(COC([*])=O)OC([*])=O | 66967 | 4 |
| Unspecific | PC(40:1) | M+Na | C48H94NO8P | 866,6609046 | SLM:000056555 | SLM:000055211 | C[N+](C)(C)CCOP([O-])(=O)OCC(COC([*])=O)OC([*])=O | 66865 | 6 |
| Unspecific | PS(40:2) | M+Na | C46H86NO10P | 866,5881336 | SLM:000058911 | SLM:000055218 | [NH3+][C@@H](COP([O-])(=O)OCC(COC([*])=O)OC([*])=O)C([O-])=O | 72080 | 9 |
| Unspecific | TG(51:3) | M+Na | C54H98O6 | 865,72554 | SLM:000308288 | SLM:000000400 | [*]C(=O)OCC(COC([*])=O)OC([*])=O | 0 | 3 |
| Unspecific | PC(42:5) | M+H | C50H90NO8P | 864,6476598 | SLM:000056579 | SLM:000055211 | C[N+](C)(C)CCOP([O-])(=O)OCC(COC([*])=O)OC([*])=O | 66966 | 7 |
| Unspecific | PC(40:2) | M+Na | C48H92NO8P | 864,6452545 | SLM:000056557 | SLM:000055211 | C[N+](C)(C)CCOP([O-])(=O)OCC(COC([*])=O)OC([*])=O | 66864 | 9 |
| Unspecific | PS(40:3) | M+Na | C46H84NO10P | 864,5724835 | SLM:000058912 | SLM:000055218 | [NH3+][C@@H](COP([O-])(=O)OCC(COC([*])=O)OC([*])=O)C([O-])=O | 72081 | 3 |
| Unspecific | TG(51:4) | M+Na | C54H96O6 | 863,7098899 | SLM:000308289 | SLM:000000400 | [*]C(=O)OCC(COC([*])=O)OC([*])=O | 0 | 3 |
| Unspecific | SM(d44:2) | M+Na | C49H97N2O6P | 863,6976244 | SLM:000390849 | SLM:000001000 | C[N+](C)(C)CCOP([O-])(=O)OC[C@H](NC([*])=O)[C@H](O)[*] | 0 | 9 |
| Unspecific | PC(42:6) | M+H | C50H88NO8P | 862,6320098 | SLM:000056580 | SLM:000055211 | C[N+](C)(C)CCOP([O-])(=O)OCC(COC([*])=O)OC([*])=O | 66965 | 7 |
| Unspecific | PC(40:3) | M+Na | C48H90NO8P | 862,6296045 | SLM:000056558 | SLM:000055211 | C[N+](C)(C)CCOP([O-])(=O)OCC(COC([*])=O)OC([*])=O | 66863 | 9 |
| Unspecific | PC(42:8) | M+H | C50H84NO8P | 858,6007096 | SLM:000056582 | SLM:000055211 | C[N+](C)(C)CCOP([O-])(=O)OCC(COC([*])=O)OC([*])=O | 0 | 5 |
| Unspecific | PC(40:5) | M+Na | C48H86NO8P | 858,5983043 | SLM:000056560 | SLM:000055211 | C[N+](C)(C)CCOP([O-])(=O)OCC(COC([*])=O)OC([*])=O | 64524 | 9 |
| Unspecific | PS(38:0) | M+K | C44H86NO10P | 858,5620708 | SLM:000058891 | SLM:000055218 | [NH3+][C@@H](COP([O-])(=O)OCC(COC([*])=O)OC([*])=O)C([O-])=O | 72071 | 9 |
| Unspecific | TG(52:3) | M+H | C55H100O6 | 857,7592454 | SLM:000308299 | SLM:000000400 | [*]C(=O)OCC(COC([*])=O)OC([*])=O | 0 | 8 |
| Unspecific | TG(50:0) | M+Na | C53H102O6 | 857,7568401 | SLM:000308275 | SLM:000000400 | [*]C(=O)OCC(COC([*])=O)OC([*])=O | 0 | 6 |
| Unspecific | PS(41:3) | M+H | C47H86NO10P | 856,6061889 | SLM:000058922 | SLM:000055218 | [NH3+][C@@H](COP([O-])(=O)OCC(COC([*])=O)OC([*])=O)C([O-])=O | 0 | 9 |
| Unspecific | PE(42:0) | M+Na | C47H94NO8P | 854,6609046 | SLM:000057230 | SLM:000055213 | [NH3+]CCOP([O-])(=O)OCC(COC([*])=O)OC([*])=O | 71747 | 4 |
| Unspecific | PC(38:1) | M+K | C46H90NO8P | 854,6035417 | SLM:000056538 | SLM:000055211 | C[N+](C)(C)CCOP([O-])(=O)OCC(COC([*])=O)OC([*])=O | 66860 | 3 |
| Unspecific | PS(41:4) | M+H | C47H84NO10P | 854,5905389 | SLM:000058923 | SLM:000055218 | [NH3+][C@@H](COP([O-])(=O)OCC(COC([*])=O)OC([*])=O)C([O-])=O | 0 | 8 |
| Unspecific | PS(39:1) | M+Na | C45H86NO10P | 854,5881336 | SLM:000058902 | SLM:000055218 | [NH3+][C@@H](COP([O-])(=O)OCC(COC([*])=O)OC([*])=O)C([O-])=O | 0 | 8 |
| Unspecific | PC(39:1) | M+Na | C47H92NO8P | 852,6452545 | SLM:000056548 | SLM:000055211 | C[N+](C)(C)CCOP([O-])(=O)OCC(COC([*])=O)OC([*])=O | 0 | 3 |
| Unspecific | PC(38:2) | M+K | C46H88NO8P | 852,5878916 | SLM:000056539 | SLM:000055211 | C[N+](C)(C)CCOP([O-])(=O)OCC(COC([*])=O)OC([*])=O | 66859 | 5 |
| Unspecific | PC(40:8) | M+Na | C48H80NO8P | 852,5513541 | SLM:000056563 | SLM:000055211 | C[N+](C)(C)CCOP([O-])(=O)OCC(COC([*])=O)OC([*])=O | 0 | 3 |
| Unspecific | TG(50:3) | M+Na | C53H96O6 | 851,7098899 | SLM:000308278 | SLM:000000400 | [*]C(=O)OCC(COC([*])=O)OC([*])=O | 0 | 9 |
| Unspecific | SM(d42:2) | M+K | C47H93N2O6P | 851,6402616 | SLM:000390823 | SLM:000001000 | C[N+](C)(C)CCOP([O-])(=O)OC[C@H](NC([*])=O)[C@H](O)[*] | 0 | 8 |
| Unspecific | PE(44:5) | M+H | C49H88NO8P | 850,6320098 | SLM:000057257 | SLM:000055213 | [NH3+]CCOP([O-])(=O)OCC(COC([*])=O)OC([*])=O | 71759 | 3 |
| Unspecific | TG(50:4) | M+Na | C53H94O6 | 849,6942398 | SLM:000308279 | SLM:000000400 | [*]C(=O)OCC(COC([*])=O)OC([*])=O | 0 | 4 |
| Unspecific | PE(42:3) | M+Na | C47H88NO8P | 848,6139544 | SLM:000057235 | SLM:000055213 | [NH3+]CCOP([O-])(=O)OCC(COC([*])=O)OC([*])=O | 71750 | 5 |
| Unspecific | PE(41:4) | M+K | C46H84NO8P | 848,5565915 | SLM:000057227 | SLM:000055213 | [NH3+]CCOP([O-])(=O)OCC(COC([*])=O)OC([*])=O | 0 | 8 |
| Unspecific | PC(40:0) | M+H | C48H96NO8P | 846,69461 | SLM:000056554 | SLM:000055211 | C[N+](C)(C)CCOP([O-])(=O)OCC(COC([*])=O)OC([*])=O | 66866 | 6 |
| Unspecific | PS(39:5) | M+Na | C45H78NO10P | 846,5255333 | SLM:000058906 | SLM:000055218 | [NH3+][C@@H](COP([O-])(=O)OCC(COC([*])=O)OC([*])=O)C([O-])=O | 0 | 4 |
| Unspecific | PE(42:5) | M+Na | C47H84NO8P | 844,5826543 | SLM:000057237 | SLM:000055213 | [NH3+]CCOP([O-])(=O)OCC(COC([*])=O)OC([*])=O | 71752 | 3 |
| Unspecific | PE(42:6) | M+Na | C47H82NO8P | 842,5670042 | SLM:000057238 | SLM:000055213 | [NH3+]CCOP([O-])(=O)OCC(COC([*])=O)OC([*])=O | 71753 | 3 |
| Unspecific | SM(d44:2) | M+H | C49H97N2O6P | 841,7156798 | SLM:000390849 | SLM:000001000 | C[N+](C)(C)CCOP([O-])(=O)OC[C@H](NC([*])=O)[C@H](O)[*] | 0 | 9 |
| Unspecific | PE(43:4) | M+H | C48H88NO8P | 838,6320098 | SLM:000057246 | SLM:000055213 | [NH3+]CCOP([O-])(=O)OCC(COC([*])=O)OC([*])=O | 0 | 5 |
| Unspecific | PC(38:1) | M+Na | C46H90NO8P | 838,6296045 | SLM:000056538 | SLM:000055211 | C[N+](C)(C)CCOP([O-])(=O)OCC(COC([*])=O)OC([*])=O | 66860 | 3 |
| Unspecific | SM(d42:1) | M+Na | C47H95N2O6P | 837,6819744 | SLM:000390824 | SLM:000001000 | C[N+](C)(C)CCOP([O-])(=O)OC[C@H](NC([*])=O)[C@H](O)[*] | 0 | 9 |
| Unspecific | PC(40:5) | M+H | C48H86NO8P | 836,6163597 | SLM:000056560 | SLM:000055211 | C[N+](C)(C)CCOP([O-])(=O)OCC(COC([*])=O)OC([*])=O | 64524 | 9 |
| Unspecific | PC(38:2) | M+Na | C46H88NO8P | 836,6139544 | SLM:000056539 | SLM:000055211 | C[N+](C)(C)CCOP([O-])(=O)OCC(COC([*])=O)OC([*])=O | 66859 | 5 |
| Unspecific | SM(d42:2) | M+Na | C47H93N2O6P | 835,6663243 | SLM:000390823 | SLM:000001000 | C[N+](C)(C)CCOP([O-])(=O)OC[C@H](NC([*])=O)[C@H](O)[*] | 0 | 8 |
| Unspecific | PC(40:8) | M+H | C48H80NO8P | 830,5694095 | SLM:000056563 | SLM:000055211 | C[N+](C)(C)CCOP([O-])(=O)OCC(COC([*])=O)OC([*])=O | 0 | 3 |
| Unspecific | PE(41:5) | M+Na | C46H82NO8P | 830,5670042 | SLM:000057228 | SLM:000055213 | [NH3+]CCOP([O-])(=O)OCC(COC([*])=O)OC([*])=O | 0 | 9 |
| Unspecific | PS(36:0) | M+K | C42H82NO10P | 830,5307707 | SLM:000058875 | SLM:000055218 | [NH3+][C@@H](COP([O-])(=O)OCC(COC([*])=O)OC([*])=O)C([O-])=O | 72064 | 9 |
| Unspecific | TG(50:3) | M+H | C53H96O6 | 829,7279453 | SLM:000308278 | SLM:000000400 | [*]C(=O)OCC(COC([*])=O)OC([*])=O | 0 | 9 |
| Unspecific | TG(48:0) | M+Na | C51H98O6 | 829,72554 | SLM:000308257 | SLM:000000400 | [*]C(=O)OCC(COC([*])=O)OC([*])=O | 0 | 6 |
| Unspecific | PE(41:6) | M+Na | C46H80NO8P | 828,5513541 | SLM:000057229 | SLM:000055213 | [NH3+]CCOP([O-])(=O)OCC(COC([*])=O)OC([*])=O | 0 | 5 |
| Unspecific | TG(50:4) | M+H | C53H94O6 | 827,7122952 | SLM:000308279 | SLM:000000400 | [*]C(=O)OCC(COC([*])=O)OC([*])=O | 0 | 4 |
| Unspecific | TG(48:1) | M+Na | C51H96O6 | 827,7098899 | SLM:000308258 | SLM:000000400 | [*]C(=O)OCC(COC([*])=O)OC([*])=O | 0 | 4 |
| Unspecific | PS(37:1) | M+Na | C43H82NO10P | 826,5568334 | SLM:000058885 | SLM:000055218 | [NH3+][C@@H](COP([O-])(=O)OCC(COC([*])=O)OC([*])=O)C([O-])=O | 0 | 3 |
| Unspecific | PE(42:4) | M+H | C47H86NO8P | 824,6163597 | SLM:000057236 | SLM:000055213 | [NH3+]CCOP([O-])(=O)OCC(COC([*])=O)OC([*])=O | 71751 | 9 |
| Unspecific | PC(37:1) | M+Na | C45H88NO8P | 824,6139544 | SLM:000056531 | SLM:000055211 | C[N+](C)(C)CCOP([O-])(=O)OCC(COC([*])=O)OC([*])=O | 0 | 9 |
| Unspecific | PC(36:2) | M+K | C44H84NO8P | 824,5565915 | SLM:000056523 | SLM:000055211 | C[N+](C)(C)CCOP([O-])(=O)OCC(COC([*])=O)OC([*])=O | 64433 | 9 |
| Unspecific | SM(d41:1) | M+Na | C46H93N2O6P | 823,6663243 | SLM:000390811 | SLM:000001000 | C[N+](C)(C)CCOP([O-])(=O)OC[C@H](NC([*])=O)[C@H](O)[*] | 0 | 9 |
| Unspecific | SM(d40:2) | M+K | C45H89N2O6P | 823,6089614 | SLM:000390795 | SLM:000001000 | C[N+](C)(C)CCOP([O-])(=O)OC[C@H](NC([*])=O)[C@H](O)[*] | 0 | 8 |
| Unspecific | SM(d40:3) | M+K | C45H87N2O6P | 821,5933114 | SLM:000390793 | SLM:000001000 | C[N+](C)(C)CCOP([O-])(=O)OC[C@H](NC([*])=O)[C@H](O)[*] | 0 | 9 |
| Unspecific | PS(37:4) | M+Na | C43H76NO10P | 820,5098832 | SLM:000058888 | SLM:000055218 | [NH3+][C@@H](COP([O-])(=O)OCC(COC([*])=O)OC([*])=O)C([O-])=O | 0 | 5 |
| Unspecific | PE(42:7) | M+H | C47H80NO8P | 818,5694095 | SLM:000057239 | SLM:000055213 | [NH3+]CCOP([O-])(=O)OCC(COC([*])=O)OC([*])=O | 0 | 5 |
| Unspecific | PE(40:4) | M+Na | C45H82NO8P | 818,5670042 | SLM:000057217 | SLM:000055213 | [NH3+]CCOP([O-])(=O)OCC(COC([*])=O)OC([*])=O | 71744 | 9 |
| Unspecific | PC(38:1) | M+H | C46H90NO8P | 816,6476598 | SLM:000056538 | SLM:000055211 | C[N+](C)(C)CCOP([O-])(=O)OCC(COC([*])=O)OC([*])=O | 66860 | 3 |
| Unspecific | SM(d42:1) | M+H | C47H95N2O6P | 815,7000298 | SLM:000390824 | SLM:000001000 | C[N+](C)(C)CCOP([O-])(=O)OC[C@H](NC([*])=O)[C@H](O)[*] | 0 | 9 |
| Unspecific | PC(38:2) | M+H | C46H88NO8P | 814,6320098 | SLM:000056539 | SLM:000055211 | C[N+](C)(C)CCOP([O-])(=O)OCC(COC([*])=O)OC([*])=O | 66859 | 5 |
| Unspecific | PS(38:3) | M+H | C44H80NO10P | 814,5592387 | SLM:000058894 | SLM:000055218 | [NH3+][C@@H](COP([O-])(=O)OCC(COC([*])=O)OC([*])=O)C([O-])=O | 72074 | 7 |
| Unspecific | PS(36:0) | M+Na | C42H82NO10P | 814,5568334 | SLM:000058875 | SLM:000055218 | [NH3+][C@@H](COP([O-])(=O)OCC(COC([*])=O)OC([*])=O)C([O-])=O | 72064 | 9 |
| Unspecific | SM(d42:2) | M+H | C47H93N2O6P | 813,6843797 | SLM:000390823 | SLM:000001000 | C[N+](C)(C)CCOP([O-])(=O)OC[C@H](NC([*])=O)[C@H](O)[*] | 0 | 8 |
| Unspecific | PE(41:3) | M+H | C46H86NO8P | 812,6163597 | SLM:000057226 | SLM:000055213 | [NH3+]CCOP([O-])(=O)OCC(COC([*])=O)OC([*])=O | 0 | 5 |
| Unspecific | PC(36:0) | M+Na | C44H88NO8P | 812,6139544 | SLM:000056521 | SLM:000055211 | C[N+](C)(C)CCOP([O-])(=O)OCC(COC([*])=O)OC([*])=O | 66858 | 9 |
| Unspecific | PE(41:4) | M+H | C46H84NO8P | 810,6007096 | SLM:000057227 | SLM:000055213 | [NH3+]CCOP([O-])(=O)OCC(COC([*])=O)OC([*])=O | 0 | 8 |
| Unspecific | PE(39:1) | M+Na | C44H86NO8P | 810,5983043 | SLM:000057206 | SLM:000055213 | [NH3+]CCOP([O-])(=O)OCC(COC([*])=O)OC([*])=O | 0 | 9 |
| Unspecific | SM(d40:1) | M+Na | C45H91N2O6P | 809,6506743 | SLM:000390797 | SLM:000001000 | C[N+](C)(C)CCOP([O-])(=O)OC[C@H](NC([*])=O)[C@H](O)[*] | 0 | 9 |
| Unspecific | PE(41:5) | M+H | C46H82NO8P | 808,5850596 | SLM:000057228 | SLM:000055213 | [NH3+]CCOP([O-])(=O)OCC(COC([*])=O)OC([*])=O | 0 | 9 |
| Unspecific | PC(36:2) | M+Na | C44H84NO8P | 808,5826543 | SLM:000056523 | SLM:000055211 | C[N+](C)(C)CCOP([O-])(=O)OCC(COC([*])=O)OC([*])=O | 64433 | 9 |
| Unspecific | SM(d40:2) | M+Na | C45H89N2O6P | 807,6350242 | SLM:000390795 | SLM:000001000 | C[N+](C)(C)CCOP([O-])(=O)OC[C@H](NC([*])=O)[C@H](O)[*] | 0 | 8 |
| Unspecific | PE(41:6) | M+H | C46H80NO8P | 806,5694095 | SLM:000057229 | SLM:000055213 | [NH3+]CCOP([O-])(=O)OCC(COC([*])=O)OC([*])=O | 0 | 5 |
| Unspecific | PE(39:3) | M+Na | C44H82NO8P | 806,5670042 | SLM:000057208 | SLM:000055213 | [NH3+]CCOP([O-])(=O)OCC(COC([*])=O)OC([*])=O | 0 | 5 |
| Unspecific | SM(d40:3) | M+Na | C45H87N2O6P | 805,6193741 | SLM:000390793 | SLM:000001000 | C[N+](C)(C)CCOP([O-])(=O)OC[C@H](NC([*])=O)[C@H](O)[*] | 0 | 9 |
| Unspecific | PE(39:4) | M+Na | C44H80NO8P | 804,5513541 | SLM:000057209 | SLM:000055213 | [NH3+]CCOP([O-])(=O)OCC(COC([*])=O)OC([*])=O | 0 | 8 |
| Unspecific | PC(37:1) | M+H | C45H88NO8P | 802,6320098 | SLM:000056531 | SLM:000055211 | C[N+](C)(C)CCOP([O-])(=O)OCC(COC([*])=O)OC([*])=O | 0 | 9 |
| Unspecific | PE(39:5) | M+Na | C44H78NO8P | 802,5357041 | SLM:000057210 | SLM:000055213 | [NH3+]CCOP([O-])(=O)OCC(COC([*])=O)OC([*])=O | 0 | 3 |
| Unspecific | PS(34:0) | M+K | C40H78NO10P | 802,4994705 | SLM:000058861 | SLM:000055218 | [NH3+][C@@H](COP([O-])(=O)OCC(COC([*])=O)OC([*])=O)C([O-])=O | 72057 | 9 |
| Unspecific | SM(d41:1) | M+H | C46H93N2O6P | 801,6843797 | SLM:000390811 | SLM:000001000 | C[N+](C)(C)CCOP([O-])(=O)OC[C@H](NC([*])=O)[C@H](O)[*] | 0 | 9 |
| Unspecific | PE(40:2) | M+H | C45H86NO8P | 800,6163597 | SLM:000057215 | SLM:000055213 | [NH3+]CCOP([O-])(=O)OCC(COC([*])=O)OC([*])=O | 71742 | 7 |
| Unspecific | PC(34:0) | M+K | C42H84NO8P | 800,5565915 | SLM:000056507 | SLM:000055211 | C[N+](C)(C)CCOP([O-])(=O)OCC(COC([*])=O)OC([*])=O | 66855 | 8 |
| Unspecific | PS(35:0) | M+Na | C41H80NO10P | 800,5411834 | SLM:000058868 | SLM:000055218 | [NH3+][C@@H](COP([O-])(=O)OCC(COC([*])=O)OC([*])=O)C([O-])=O | 0 | 8 |
| Unspecific | PE(40:3) | M+H | C45H84NO8P | 798,6007096 | SLM:000057216 | SLM:000055213 | [NH3+]CCOP([O-])(=O)OCC(COC([*])=O)OC([*])=O | 71743 | 7 |
| Unspecific | PE(38:0) | M+Na | C43H86NO8P | 798,5983043 | SLM:000057195 | SLM:000055213 | [NH3+]CCOP([O-])(=O)OCC(COC([*])=O)OC([*])=O | 71733 | 9 |
| Unspecific | PS(34:2) | M+K | C40H74NO10P | 798,4681704 | SLM:000058863 | SLM:000055218 | [NH3+][C@@H](COP([O-])(=O)OCC(COC([*])=O)OC([*])=O)C([O-])=O | 72059 | 3 |
| Unspecific | SM(d39:1) | M+Na | C44H89N2O6P | 795,6350242 | SLM:000390782 | SLM:000001000 | C[N+](C)(C)CCOP([O-])(=O)OC[C@H](NC([*])=O)[C@H](O)[*] | 0 | 9 |
| Unspecific | SM(d38:2) | M+K | C43H85N2O6P | 795,5776613 | SLM:000390765 | SLM:000001000 | C[N+](C)(C)CCOP([O-])(=O)OC[C@H](NC([*])=O)[C@H](O)[*] | 0 | 7 |
| Unspecific | PE(40:5) | M+H | C45H80NO8P | 794,5694095 | SLM:000057218 | SLM:000055213 | [NH3+]CCOP([O-])(=O)OCC(COC([*])=O)OC([*])=O | 71745 | 9 |
| Unspecific | PE(38:2) | M+Na | C43H82NO8P | 794,5670042 | SLM:000057197 | SLM:000055213 | [NH3+]CCOP([O-])(=O)OCC(COC([*])=O)OC([*])=O | 71735 | 9 |
| Unspecific | SM(d39:2) | M+Na | C44H87N2O6P | 793,6193741 | SLM:000390780 | SLM:000001000 | C[N+](C)(C)CCOP([O-])(=O)OC[C@H](NC([*])=O)[C@H](O)[*] | 0 | 9 |
| Unspecific | PE(38:3) | M+Na | C43H80NO8P | 792,5513541 | SLM:000057198 | SLM:000055213 | [NH3+]CCOP([O-])(=O)OCC(COC([*])=O)OC([*])=O | 71736 | 3 |
| Unspecific | PE(38:4) | M+Na | C43H78NO8P | 790,5357041 | SLM:000057199 | SLM:000055213 | [NH3+]CCOP([O-])(=O)OCC(COC([*])=O)OC([*])=O | 71737 | 9 |
| Unspecific | PE(39:1) | M+H | C44H86NO8P | 788,6163597 | SLM:000057206 | SLM:000055213 | [NH3+]CCOP([O-])(=O)OCC(COC([*])=O)OC([*])=O | 0 | 9 |
| Unspecific | SM(d40:1) | M+H | C45H91N2O6P | 787,6687296 | SLM:000390797 | SLM:000001000 | C[N+](C)(C)CCOP([O-])(=O)OC[C@H](NC([*])=O)[C@H](O)[*] | 0 | 9 |
| Unspecific | PC(36:2) | M+H | C44H84NO8P | 786,6007096 | SLM:000056523 | SLM:000055211 | C[N+](C)(C)CCOP([O-])(=O)OCC(COC([*])=O)OC([*])=O | 64433 | 9 |
| Unspecific | SM(d40:2) | M+H | C45H89N2O6P | 785,6530796 | SLM:000390795 | SLM:000001000 | C[N+](C)(C)CCOP([O-])(=O)OC[C@H](NC([*])=O)[C@H](O)[*] | 0 | 8 |
| Unspecific | PE(39:3) | M+H | C44H82NO8P | 784,5850596 | SLM:000057208 | SLM:000055213 | [NH3+]CCOP([O-])(=O)OCC(COC([*])=O)OC([*])=O | 0 | 5 |
| Unspecific | PC(34:0) | M+Na | C42H84NO8P | 784,5826543 | SLM:000056507 | SLM:000055211 | C[N+](C)(C)CCOP([O-])(=O)OCC(COC([*])=O)OC([*])=O | 66855 | 8 |
| Unspecific | PE(36:1) | M+K | C41H80NO8P | 784,5252914 | SLM:000057180 | SLM:000055213 | [NH3+]CCOP([O-])(=O)OCC(COC([*])=O)OC([*])=O | 71727 | 6 |
| Unspecific | SM(d40:3) | M+H | C45H87N2O6P | 783,6374295 | SLM:000390793 | SLM:000001000 | C[N+](C)(C)CCOP([O-])(=O)OC[C@H](NC([*])=O)[C@H](O)[*] | 0 | 9 |
| Unspecific | PE(39:4) | M+H | C44H80NO8P | 782,5694095 | SLM:000057209 | SLM:000055213 | [NH3+]CCOP([O-])(=O)OCC(COC([*])=O)OC([*])=O | 0 | 8 |
| Unspecific | SM(d38:1) | M+Na | C43H87N2O6P | 781,6193741 | SLM:000390767 | SLM:000001000 | C[N+](C)(C)CCOP([O-])(=O)OC[C@H](NC([*])=O)[C@H](O)[*] | 0 | 9 |
| Unspecific | PE(39:6) | M+H | C44H76NO8P | 778,5381094 | SLM:000057211 | SLM:000055213 | [NH3+]CCOP([O-])(=O)OCC(COC([*])=O)OC([*])=O | 0 | 3 |
| Unspecific | PE(37:3) | M+Na | C42H78NO8P | 778,5357041 | SLM:000057191 | SLM:000055213 | [NH3+]CCOP([O-])(=O)OCC(COC([*])=O)OC([*])=O | 0 | 9 |
| Unspecific | PE(38:0) | M+H | C43H86NO8P | 776,6163597 | SLM:000057195 | SLM:000055213 | [NH3+]CCOP([O-])(=O)OCC(COC([*])=O)OC([*])=O | 71733 | 9 |
| Unspecific | PE(38:1) | M+H | C43H84NO8P | 774,6007096 | SLM:000057196 | SLM:000055213 | [NH3+]CCOP([O-])(=O)OCC(COC([*])=O)OC([*])=O | 71734 | 4 |
| Unspecific | SM(d39:1) | M+H | C44H89N2O6P | 773,6530796 | SLM:000390782 | SLM:000001000 | C[N+](C)(C)CCOP([O-])(=O)OC[C@H](NC([*])=O)[C@H](O)[*] | 0 | 9 |
| Unspecific | PE(38:2) | M+H | C43H82NO8P | 772,5850596 | SLM:000057197 | SLM:000055213 | [NH3+]CCOP([O-])(=O)OCC(COC([*])=O)OC([*])=O | 71735 | 9 |
| Unspecific | PE(35:0) | M+K | C40H80NO8P | 772,5252914 | SLM:000057172 | SLM:000055213 | [NH3+]CCOP([O-])(=O)OCC(COC([*])=O)OC([*])=O | 0 | 9 |
| Unspecific | SM(d39:2) | M+H | C44H87N2O6P | 771,6374295 | SLM:000390780 | SLM:000001000 | C[N+](C)(C)CCOP([O-])(=O)OC[C@H](NC([*])=O)[C@H](O)[*] | 0 | 9 |
| Unspecific | PE(38:3) | M+H | C43H80NO8P | 770,5694095 | SLM:000057198 | SLM:000055213 | [NH3+]CCOP([O-])(=O)OCC(COC([*])=O)OC([*])=O | 71736 | 3 |
| Unspecific | PE(36:0) | M+Na | C41H82NO8P | 770,5670042 | SLM:000057179 | SLM:000055213 | [NH3+]CCOP([O-])(=O)OCC(COC([*])=O)OC([*])=O | 71726 | 9 |
| Unspecific | PC(32:1) | M+K | C40H78NO8P | 770,5096413 | SLM:000056494 | SLM:000055211 | C[N+](C)(C)CCOP([O-])(=O)OCC(COC([*])=O)OC([*])=O | 66849 | 7 |
| Unspecific | PE(38:4) | M+H | C43H78NO8P | 768,5537594 | SLM:000057199 | SLM:000055213 | [NH3+]CCOP([O-])(=O)OCC(COC([*])=O)OC([*])=O | 71737 | 9 |
| Unspecific | PE(36:1) | M+Na | C41H80NO8P | 768,5513541 | SLM:000057180 | SLM:000055213 | [NH3+]CCOP([O-])(=O)OCC(COC([*])=O)OC([*])=O | 71727 | 6 |
| Unspecific | SM(d37:1) | M+Na | C42H85N2O6P | 767,6037241 | SLM:000390753 | SLM:000001000 | C[N+](C)(C)CCOP([O-])(=O)OC[C@H](NC([*])=O)[C@H](O)[*] | 0 | 9 |
| Unspecific | PE(38:5) | M+H | C43H76NO8P | 766,5381094 | SLM:000057200 | SLM:000055213 | [NH3+]CCOP([O-])(=O)OCC(COC([*])=O)OC([*])=O | 71738 | 9 |
| Unspecific | PE(36:2) | M+Na | C41H78NO8P | 766,5357041 | SLM:000057181 | SLM:000055213 | [NH3+]CCOP([O-])(=O)OCC(COC([*])=O)OC([*])=O | 71728 | 9 |
| Unspecific | SM(d37:2) | M+Na | C42H83N2O6P | 765,588074 | SLM:000390751 | SLM:000001000 | C[N+](C)(C)CCOP([O-])(=O)OC[C@H](NC([*])=O)[C@H](O)[*] | 0 | 7 |
| Unspecific | PE(36:3) | M+Na | C41H76NO8P | 764,520054 | SLM:000057182 | SLM:000055213 | [NH3+]CCOP([O-])(=O)OCC(COC([*])=O)OC([*])=O | 71729 | 9 |
| Unspecific | PC(34:0) | M+H | C42H84NO8P | 762,6007096 | SLM:000056507 | SLM:000055211 | C[N+](C)(C)CCOP([O-])(=O)OCC(COC([*])=O)OC([*])=O | 66855 | 8 |
| Unspecific | SM(d38:1) | M+H | C43H87N2O6P | 759,6374295 | SLM:000390767 | SLM:000001000 | C[N+](C)(C)CCOP([O-])(=O)OC[C@H](NC([*])=O)[C@H](O)[*] | 0 | 9 |
| Unspecific | PE(37:2) | M+H | C42H80NO8P | 758,5694095 | SLM:000057190 | SLM:000055213 | [NH3+]CCOP([O-])(=O)OCC(COC([*])=O)OC([*])=O | 0 | 9 |
| Unspecific | PE(34:0) | M+K | C39H78NO8P | 758,5096413 | SLM:000057165 | SLM:000055213 | [NH3+]CCOP([O-])(=O)OCC(COC([*])=O)OC([*])=O | 71718 | 5 |
| Unspecific | SM(d38:2) | M+H | C43H85N2O6P | 757,6217794 | SLM:000390765 | SLM:000001000 | C[N+](C)(C)CCOP([O-])(=O)OC[C@H](NC([*])=O)[C@H](O)[*] | 0 | 7 |
| Unspecific | PE(37:3) | M+H | C42H78NO8P | 756,5537594 | SLM:000057191 | SLM:000055213 | [NH3+]CCOP([O-])(=O)OCC(COC([*])=O)OC([*])=O | 0 | 9 |
| Unspecific | PE(35:0) | M+Na | C40H80NO8P | 756,5513541 | SLM:000057172 | SLM:000055213 | [NH3+]CCOP([O-])(=O)OCC(COC([*])=O)OC([*])=O | 0 | 9 |
| Unspecific | PE(37:4) | M+H | C42H76NO8P | 754,5381094 | SLM:000057192 | SLM:000055213 | [NH3+]CCOP([O-])(=O)OCC(COC([*])=O)OC([*])=O | 0 | 9 |
| Unspecific | PC(32:1) | M+Na | C40H78NO8P | 754,5357041 | SLM:000056494 | SLM:000055211 | C[N+](C)(C)CCOP([O-])(=O)OCC(COC([*])=O)OC([*])=O | 66849 | 7 |
| Unspecific | SM(d36:1) | M+Na | C41H83N2O6P | 753,588074 | SLM:000390739 | SLM:000001000 | C[N+](C)(C)CCOP([O-])(=O)OC[C@H](NC([*])=O)[C@H](O)[*] | 0 | 9 |
| Unspecific | PE(35:2) | M+Na | C40H76NO8P | 752,520054 | SLM:000057174 | SLM:000055213 | [NH3+]CCOP([O-])(=O)OCC(COC([*])=O)OC([*])=O | 0 | 6 |
| Unspecific | SM(d36:2) | M+Na | C41H81N2O6P | 751,5724239 | SLM:000390737 | SLM:000001000 | C[N+](C)(C)CCOP([O-])(=O)OC[C@H](NC([*])=O)[C@H](O)[*] | 0 | 9 |
| Unspecific | SM(d36:3) | M+Na | C41H79N2O6P | 749,5567739 | SLM:000390735 | SLM:000001000 | C[N+](C)(C)CCOP([O-])(=O)OC[C@H](NC([*])=O)[C@H](O)[*] | 0 | 7 |
| Unspecific | PE(36:0) | M+H | C41H82NO8P | 748,5850596 | SLM:000057179 | SLM:000055213 | [NH3+]CCOP([O-])(=O)OCC(COC([*])=O)OC([*])=O | 71726 | 9 |
| Unspecific | PE(36:1) | M+H | C41H80NO8P | 746,5694095 | SLM:000057180 | SLM:000055213 | [NH3+]CCOP([O-])(=O)OCC(COC([*])=O)OC([*])=O | 71727 | 6 |
| Unspecific | SM(d37:1) | M+H | C42H85N2O6P | 745,6217794 | SLM:000390753 | SLM:000001000 | C[N+](C)(C)CCOP([O-])(=O)OC[C@H](NC([*])=O)[C@H](O)[*] | 0 | 9 |
| Unspecific | SM(d37:2) | M+H | C42H83N2O6P | 743,6061294 | SLM:000390751 | SLM:000001000 | C[N+](C)(C)CCOP([O-])(=O)OC[C@H](NC([*])=O)[C@H](O)[*] | 0 | 7 |
| Unspecific | PE(36:3) | M+H | C41H76NO8P | 742,5381094 | SLM:000057182 | SLM:000055213 | [NH3+]CCOP([O-])(=O)OCC(COC([*])=O)OC([*])=O | 71729 | 9 |
| Unspecific | PE(34:0) | M+Na | C39H78NO8P | 742,5357041 | SLM:000057165 | SLM:000055213 | [NH3+]CCOP([O-])(=O)OCC(COC([*])=O)OC([*])=O | 71718 | 5 |
| Unspecific | PE(34:1) | M+Na | C39H76NO8P | 740,520054 | SLM:000057166 | SLM:000055213 | [NH3+]CCOP([O-])(=O)OCC(COC([*])=O)OC([*])=O | 71720 | 4 |
| Unspecific | SM(d35:1) | M+Na | C40H81N2O6P | 739,5724239 | SLM:000390726 | SLM:000001000 | C[N+](C)(C)CCOP([O-])(=O)OC[C@H](NC([*])=O)[C@H](O)[*] | 0 | 9 |
| Unspecific | PE(34:2) | M+Na | C39H74NO8P | 738,5044039 | SLM:000057167 | SLM:000055213 | [NH3+]CCOP([O-])(=O)OCC(COC([*])=O)OC([*])=O | 71721 | 5 |
| Unspecific | PE(34:3) | M+Na | C39H72NO8P | 736,4887539 | SLM:000057168 | SLM:000055213 | [NH3+]CCOP([O-])(=O)OCC(COC([*])=O)OC([*])=O | 71722 | 3 |
| Unspecific | PC(32:1) | M+H | C40H78NO8P | 732,5537594 | SLM:000056494 | SLM:000055211 | C[N+](C)(C)CCOP([O-])(=O)OCC(COC([*])=O)OC([*])=O | 66849 | 7 |
| Unspecific | SM(d36:1) | M+H | C41H83N2O6P | 731,6061294 | SLM:000390739 | SLM:000001000 | C[N+](C)(C)CCOP([O-])(=O)OC[C@H](NC([*])=O)[C@H](O)[*] | 0 | 9 |
| Unspecific | PE(35:2) | M+H | C40H76NO8P | 730,5381094 | SLM:000057174 | SLM:000055213 | [NH3+]CCOP([O-])(=O)OCC(COC([*])=O)OC([*])=O | 0 | 6 |
| Unspecific | SM(d36:2) | M+H | C41H81N2O6P | 729,5904793 | SLM:000390737 | SLM:000001000 | C[N+](C)(C)CCOP([O-])(=O)OC[C@H](NC([*])=O)[C@H](O)[*] | 0 | 9 |
| Unspecific | PC(30:0) | M+Na | C38H76NO8P | 728,520054 | SLM:000056479 | SLM:000055211 | C[N+](C)(C)CCOP([O-])(=O)OCC(COC([*])=O)OC([*])=O | 65303 | 9 |
| Unspecific | SM(d36:3) | M+H | C41H79N2O6P | 727,5748292 | SLM:000390735 | SLM:000001000 | C[N+](C)(C)CCOP([O-])(=O)OC[C@H](NC([*])=O)[C@H](O)[*] | 0 | 7 |
| Unspecific | SM(d33:1) | M+K | C38H77N2O6P | 727,515061 | SLM:000390704 | SLM:000001000 | C[N+](C)(C)CCOP([O-])(=O)OC[C@H](NC([*])=O)[C@H](O)[*] | 0 | 9 |
| Unspecific | PE(33:1) | M+Na | C38H74NO8P | 726,5044039 | SLM:000057159 | SLM:000055213 | [NH3+]CCOP([O-])(=O)OCC(COC([*])=O)OC([*])=O | 0 | 8 |
| Unspecific | PE(34:1) | M+H | C39H76NO8P | 718,5381094 | SLM:000057166 | SLM:000055213 | [NH3+]CCOP([O-])(=O)OCC(COC([*])=O)OC([*])=O | 71720 | 4 |
| Unspecific | SM(d35:1) | M+H | C40H81N2O6P | 717,5904793 | SLM:000390726 | SLM:000001000 | C[N+](C)(C)CCOP([O-])(=O)OC[C@H](NC([*])=O)[C@H](O)[*] | 0 | 9 |
| Unspecific | PE(34:2) | M+H | C39H74NO8P | 716,5224593 | SLM:000057167 | SLM:000055213 | [NH3+]CCOP([O-])(=O)OCC(COC([*])=O)OC([*])=O | 71721 | 5 |
| Unspecific | SM(d33:1) | M+Na | C38H77N2O6P | 711,5411238 | SLM:000390704 | SLM:000001000 | C[N+](C)(C)CCOP([O-])(=O)OC[C@H](NC([*])=O)[C@H](O)[*] | 0 | 9 |
| Unspecific | PC(28:0) | M+Na | C36H72NO8P | 700,4887539 | SLM:000056466 | SLM:000055211 | C[N+](C)(C)CCOP([O-])(=O)OCC(COC([*])=O)OC([*])=O | 65294 | 3 |
| Unspecific | SM(d33:1) | M+H | C38H77N2O6P | 689,5591792 | SLM:000390704 | SLM:000001000 | C[N+](C)(C)CCOP([O-])(=O)OC[C@H](NC([*])=O)[C@H](O)[*] | 0 | 9 |
| Unspecific | PC(28:0) | M+H | C36H72NO8P | 678,5068092 | SLM:000056466 | SLM:000055211 | C[N+](C)(C)CCOP([O-])(=O)OCC(COC([*])=O)OC([*])=O | 65294 | 3 |
| Unspecific | DG(40:4) | M+H | C43H76O5 | 673,57653 | SLM:000307449 | SLM:000000401 | [*]OCC(CO[*])O[*] | 0 | 3 |
| Unspecific | DG(40:5) | M+H | C43H74O5 | 671,56088 | SLM:000307450 | SLM:000000401 | [*]OCC(CO[*])O[*] | 0 | 3 |
| Unspecific | DG(38:2) | M+Na | C41H76O5 | 671,5584746 | SLM:000307429 | SLM:000000401 | [*]OCC(CO[*])O[*] | 0 | 3 |
| Unspecific | DG(38:4) | M+Na | C41H72O5 | 667,5271745 | SLM:000307431 | SLM:000000401 | [*]OCC(CO[*])O[*] | 0 | 3 |
| Unspecific | DG(38:4) | M+H | C41H72O5 | 645,5452299 | SLM:000307431 | SLM:000000401 | [*]OCC(CO[*])O[*] | 0 | 3 |
| Unspecific | DG(36:1) | M+Na | C39H74O5 | 645,5428246 | SLM:000307412 | SLM:000000401 | [*]OCC(CO[*])O[*] | 0 | 3 |
| Unspecific | LPC(24:1) | M+Na | C32H64NO7P | 628,431239 | SLM:000055343 | SLM:000055200 | C[N+](C)(C)CCOP([O-])(=O)OCC(CO[*])O[*] | 74471 | 5 |
| Unspecific | DG(36:3) | M+H | C39H70O5 | 619,5295798 | SLM:000307414 | SLM:000000401 | [*]OCC(CO[*])O[*] | 0 | 4 |
| Unspecific | DG(34:1) | M+Na | C37H70O5 | 617,5115245 | SLM:000307398 | SLM:000000401 | [*]OCC(CO[*])O[*] | 0 | 3 |
| Unspecific | DG(34:2) | M+Na | C37H68O5 | 615,4958744 | SLM:000307399 | SLM:000000401 | [*]OCC(CO[*])O[*] | 0 | 3 |
| Unspecific | LPC(22:6) | M+K | C30H50NO7P | 606,2956258 | SLM:000055341 | SLM:000055200 | C[N+](C)(C)CCOP([O-])(=O)OCC(CO[*])O[*] | 0 | 6 |
| Unspecific | LPC(22:4) | M+Na | C30H54NO7P | 594,3529887 | SLM:000055339 | SLM:000055200 | C[N+](C)(C)CCOP([O-])(=O)OCC(CO[*])O[*] | 0 | 6 |
| Unspecific | LPC(22:0) | M+H | C30H62NO7P | 580,4336443 | SLM:000055335 | SLM:000055200 | C[N+](C)(C)CCOP([O-])(=O)OCC(CO[*])O[*] | 67061 | 3 |
| Unspecific | LPC(22:1) | M+H | C30H60NO7P | 578,4179942 | SLM:000055336 | SLM:000055200 | C[N+](C)(C)CCOP([O-])(=O)OCC(CO[*])O[*] | 67060 | 4 |
| Unspecific | LPC(20:0) | M+Na | C28H58NO7P | 574,3842888 | SLM:000055328 | SLM:000055200 | C[N+](C)(C)CCOP([O-])(=O)OCC(CO[*])O[*] | 67058 | 4 |
| Unspecific | LPC(22:6) | M+H | C30H50NO7P | 568,3397439 | SLM:000055341 | SLM:000055200 | C[N+](C)(C)CCOP([O-])(=O)OCC(CO[*])O[*] | 0 | 6 |
| Unspecific | Cer(d36:1) | M+H | C36H71NO3 | 566,5506496 | SLM:000391261 | SLM:000399814 | OC[C@H](NC([*])=O)[C@H](O)[*] | 0 | 3 |
| Unspecific | LPC(18:0) | M+K | C26H54NO7P | 562,3269259 | SLM:000055322 | SLM:000055200 | C[N+](C)(C)CCOP([O-])(=O)OCC(CO[*])O[*] | 64561 | 9 |
| Unspecific | Cer(d34:1) | M+Na | C34H67NO3 | 560,5012941 | SLM:000391236 | SLM:000399814 | OC[C@H](NC([*])=O)[C@H](O)[*] | 0 | 3 |
| Unspecific | LPC(20:3) | M+H | C28H52NO7P | 546,355394 | SLM:000055331 | SLM:000055200 | C[N+](C)(C)CCOP([O-])(=O)OCC(CO[*])O[*] | 0 | 6 |
| Unspecific | LPC(18:0) | M+Na | C26H54NO7P | 546,3529887 | SLM:000055322 | SLM:000055200 | C[N+](C)(C)CCOP([O-])(=O)OCC(CO[*])O[*] | 64561 | 9 |
| Unspecific | LPC(20:4) | M+H | C28H50NO7P | 544,3397439 | SLM:000055332 | SLM:000055200 | C[N+](C)(C)CCOP([O-])(=O)OCC(CO[*])O[*] | 0 | 7 |
| Unspecific | LPC(18:1) | M+Na | C26H52NO7P | 544,3373386 | SLM:000055323 | SLM:000055200 | C[N+](C)(C)CCOP([O-])(=O)OCC(CO[*])O[*] | 64566 | 9 |
| Unspecific | LPC(19:0) | M+H | C27H56NO7P | 538,3866941 | SLM:000055327 | SLM:000055200 | C[N+](C)(C)CCOP([O-])(=O)OCC(CO[*])O[*] | 0 | 6 |
| Unspecific | LPC(16:0) | M+K | C24H50NO7P | 534,2956258 | SLM:000055318 | SLM:000055200 | C[N+](C)(C)CCOP([O-])(=O)OCC(CO[*])O[*] | 64563 | 9 |
| Unspecific | LPC(17:0) | M+Na | C25H52NO7P | 532,3373386 | SLM:000055321 | SLM:000055200 | C[N+](C)(C)CCOP([O-])(=O)OCC(CO[*])O[*] | 0 | 9 |
| Unspecific | LPC(18:0) | M+H | C26H54NO7P | 524,3710441 | SLM:000055322 | SLM:000055200 | C[N+](C)(C)CCOP([O-])(=O)OCC(CO[*])O[*] | 64561 | 9 |
| Unspecific | LPC(18:2) | M+H | C26H50NO7P | 520,3397439 | SLM:000055324 | SLM:000055200 | C[N+](C)(C)CCOP([O-])(=O)OCC(CO[*])O[*] | 0 | 9 |
| Unspecific | LPC(16:0) | M+Na | C24H50NO7P | 518,3216886 | SLM:000055318 | SLM:000055200 | C[N+](C)(C)CCOP([O-])(=O)OCC(CO[*])O[*] | 64563 | 9 |
| Unspecific | LPC(15:0) | M+Na | C23H48NO7P | 504,3060385 | SLM:000055317 | SLM:000055200 | C[N+](C)(C)CCOP([O-])(=O)OCC(CO[*])O[*] | 0 | 4 |
| Unspecific | LPC(17:0) | M+H | C25H52NO7P | 510,355394 | SLM:000055321 | SLM:000055200 | C[N+](C)(C)CCOP([O-])(=O)OCC(CO[*])O[*] | 0 | 9 |
