# Supplementary figures and images for: Tissue- and sex-specific lipidomic analysis of Schistosoma mansoni using high-resolution atmospheric pressure scanning microprobe matrix-assisted laser desorption/ionization mass spectrometry imaging
Source: PLoS Negl Trop Dis. 2020 May 13;14(5):e0008145. doi: 10.1371/journal.pntd.0008145 (PMC7250470; doi:10.1371/journal.pntd.0008145)

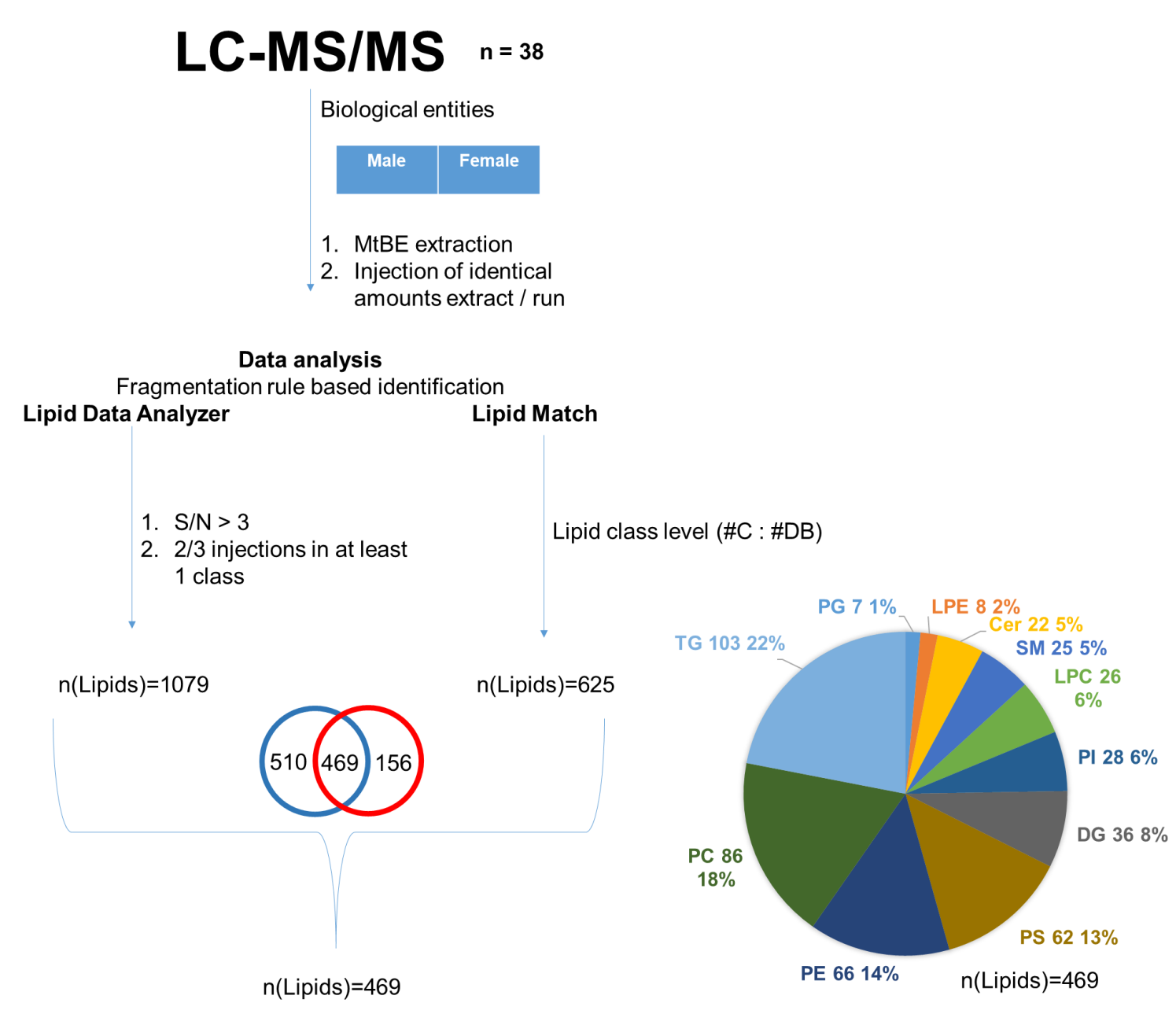

Supplement: S1 Fig — (TIF) [file pntd.0008145.s005.tif]

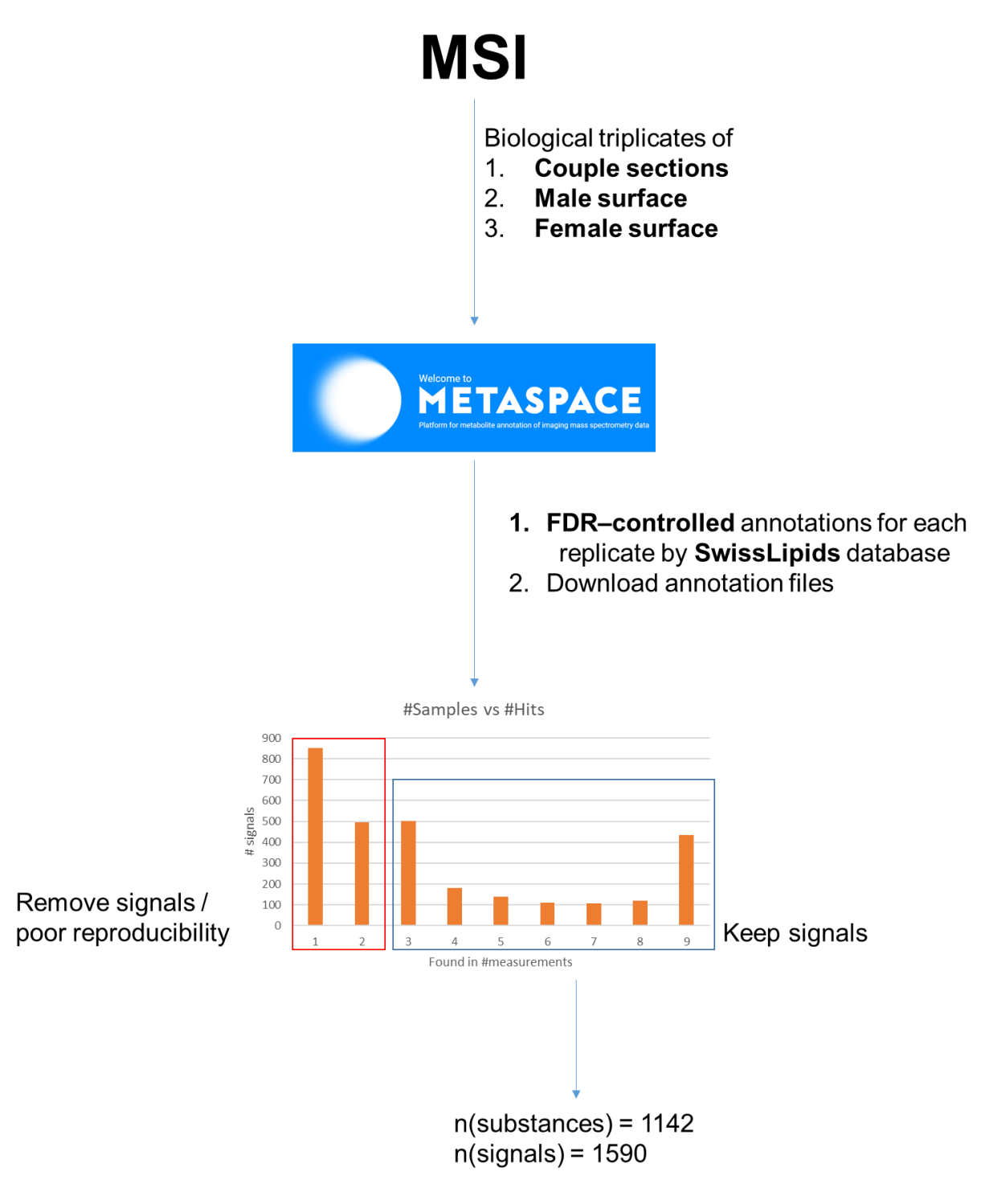

Supplement: S2 Fig — Statistical evaluation work flow was adapted from literature. The statistical analysis comprised five key steps: 1. normalization of one signal to the sum of all signals per measurement, 2. z-score (using median), 3. multiple-class analysis of variance (ANOVA, permutation based false-discovery-rate, FDR, set to 5%, 250 restarts), 4. post-hoc test (5% FDR) and 5. hierarchical clustering (Euclidean distance using average linkage, preprocessing with k-means, maximum 10 iterations, 10 restarts). (TIF) [file pntd.0008145.s006.tif]

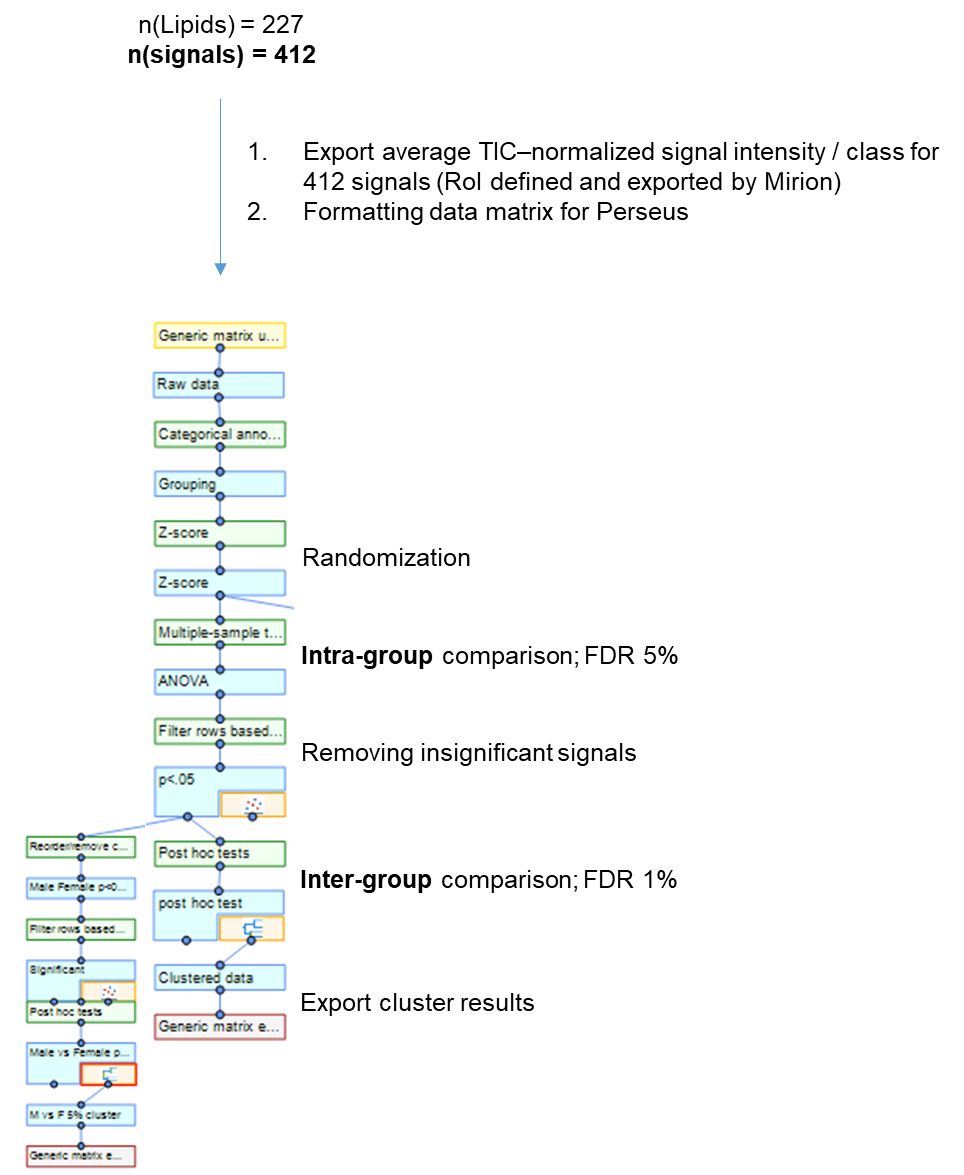

Supplement: S3 Fig — (TIF) [file pntd.0008145.s007.tif]

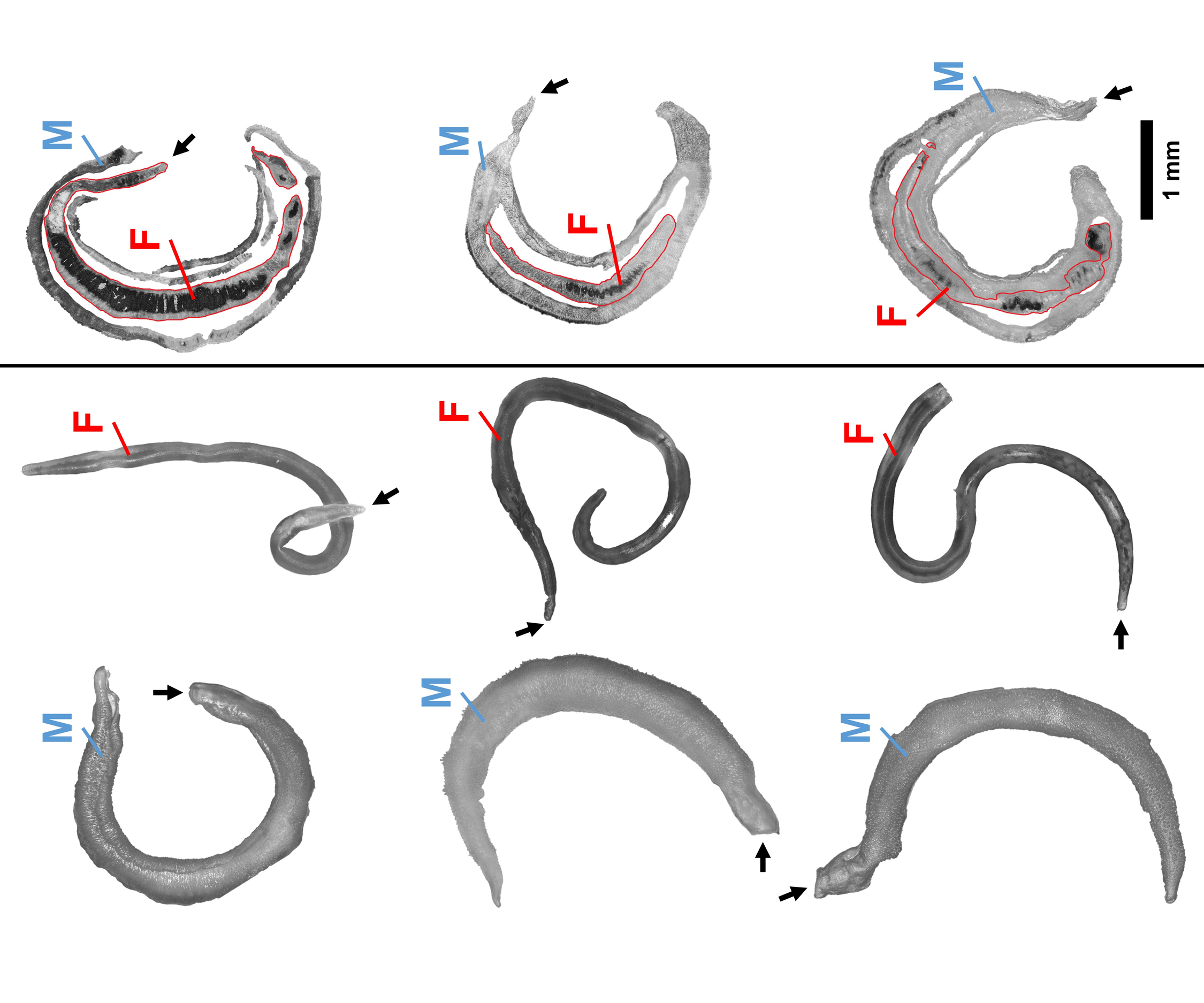

Supplement: S4 Fig — Digital light microscopic images of male (M) surfaces (left), female (F) surfaces (middle) and cryosections of couples (right). The black arrows indicate the anterior end. (TIF) [file pntd.0008145.s008.tif]

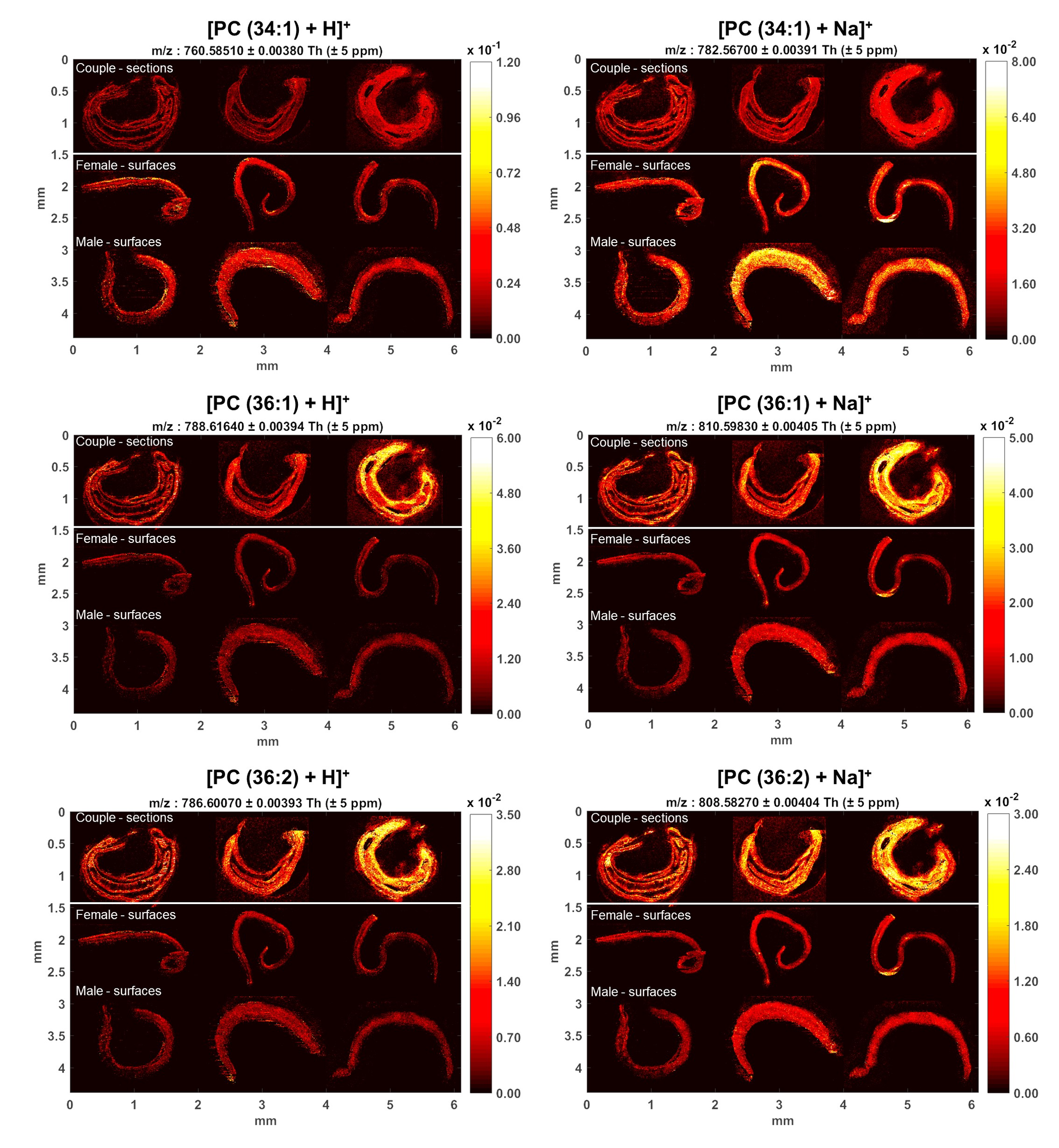

Supplement: S5 Fig — [10] However, MS imaging data did not show significant differences based on HC. For PC (36:1) and PC (36:2), however, our findings are well in accordance with previous publications which found higher abundances inside the worm.[10] The same trend is suggested by unsupervised MS imaging data evaluation presented here. (TIF) [file pntd.0008145.s009.tif]

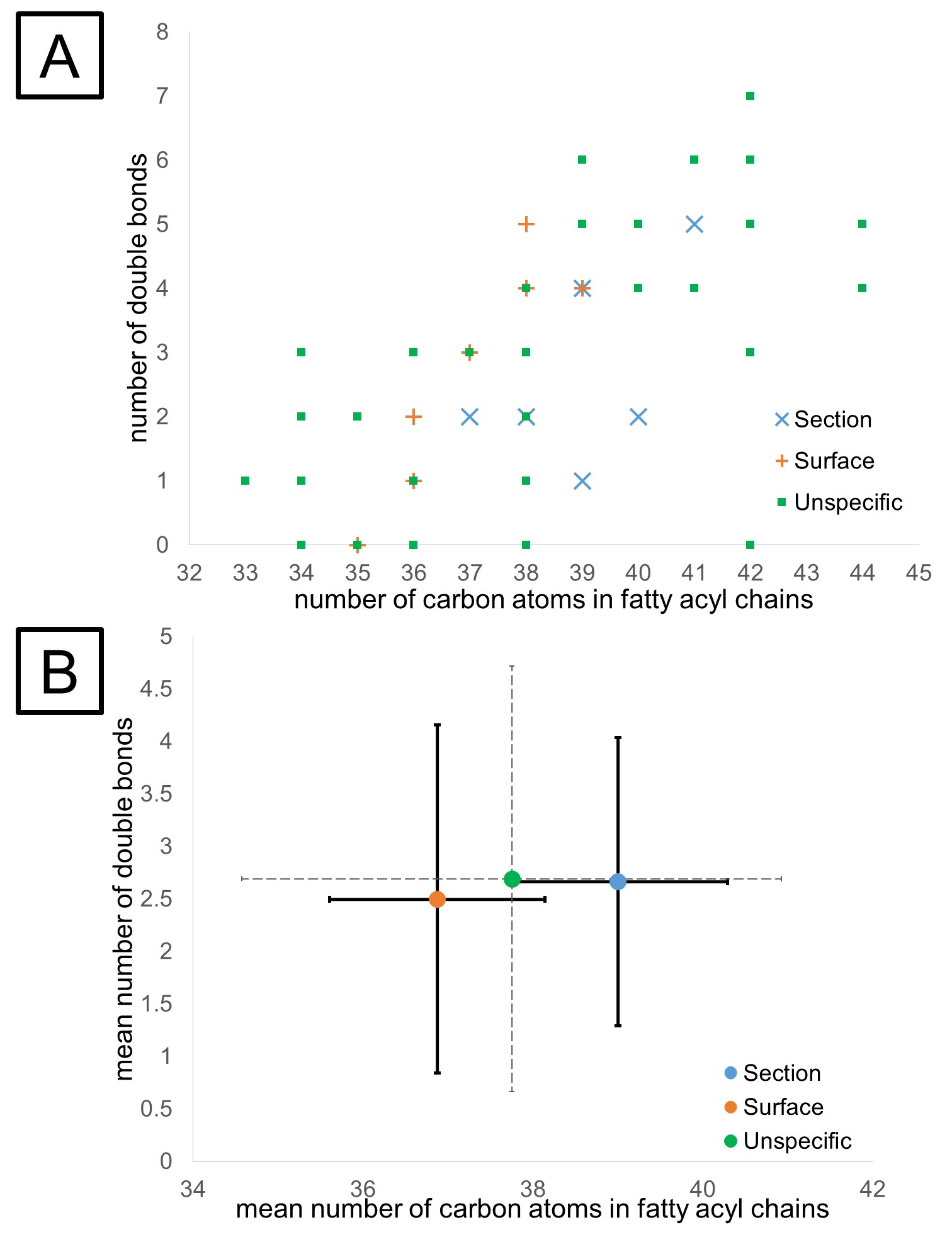

Supplement: S6 Fig — Isobaric PE/PC interferences were excluded for surface and section data. A–Comparison of worm-tissue (blue cross) and surface/tegument specific signals vs ions (orange +) with unspecific distribution (green square). Overlapping indicators are attributed to the presence of several adducts corresponding to one lipid species. B–Arithmetic mean fatty acyl and double bond composition for section/inner tissue (blue), surface/tegument (orange) and unspecific signals (green). Error bars show the standard deviation across one location. (TIF) [file pntd.0008145.s010.tif]

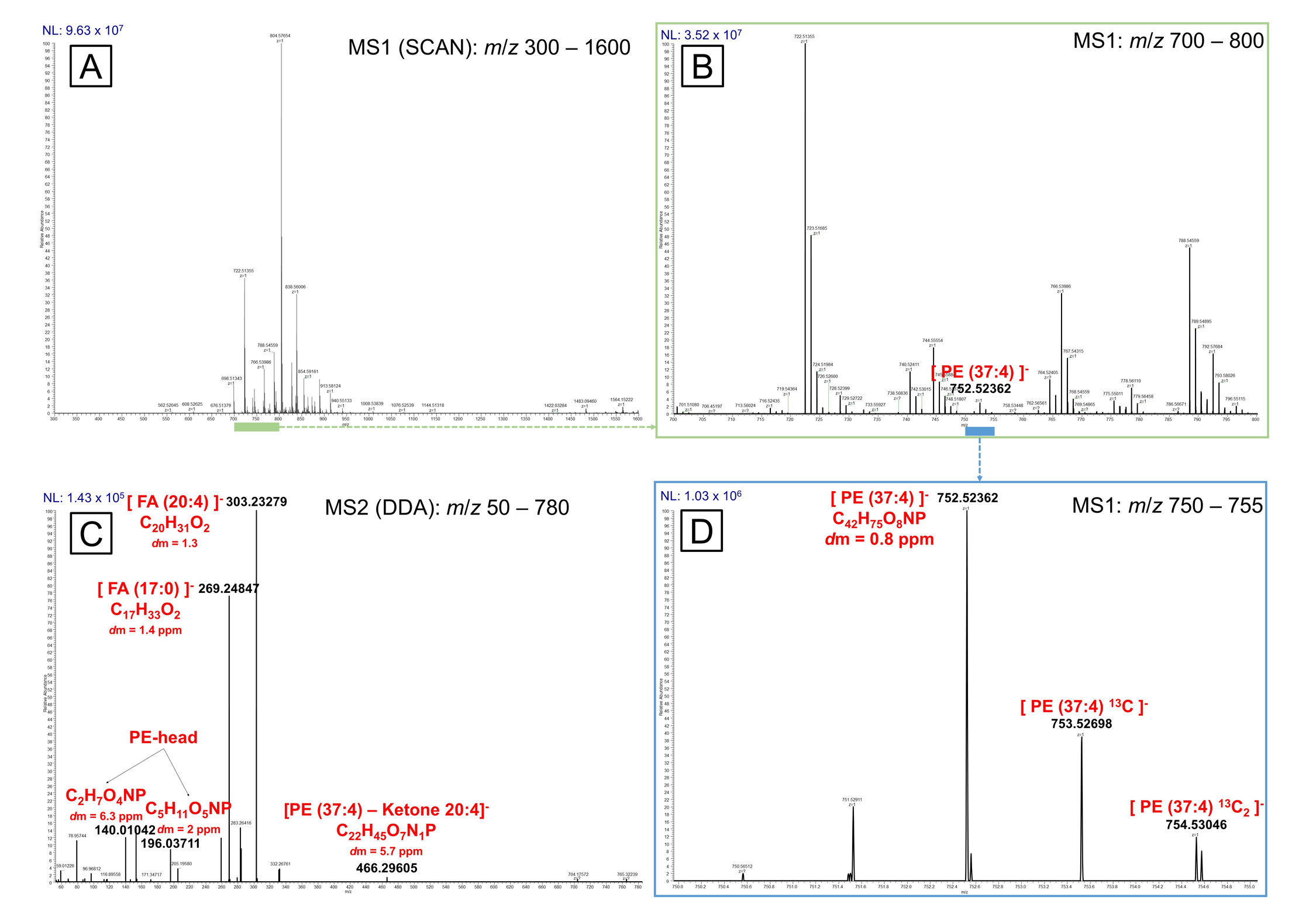

Supplement: S7 Fig — A–MS1 overview spectrum. B–virtual magnification of mass range m/z 700–800 (from A) showing the mass of PE (37:4) as deprotonated species (C42H75NO8P). C–MS2 spectrum of precursor m/z 752.52 ±0.5 u showing characteristic fragments of PE head group (around m/z 140 and m/z 196), FA (17:0) and FA (20:4). The precursor is not visible in the spectrum and assumedly fragmented quantitatively at NCE = 30. D–virtual magnification of m/z 750–755 (from A) showing mass and isotope ratio of PE 37:4 as 12C, 13C and 13C2 isotopologues. (TIF) [file pntd.0008145.s011.tif]

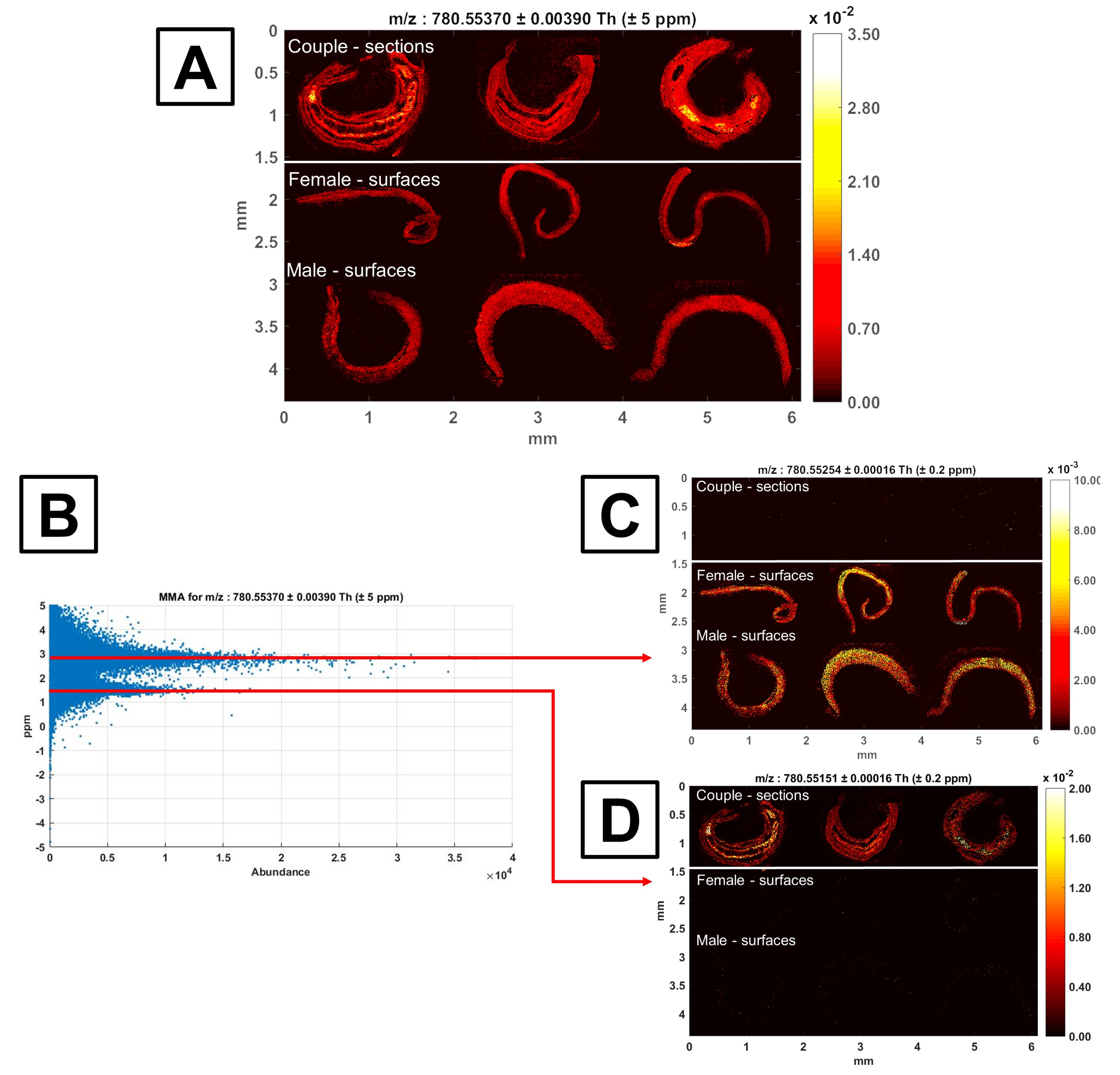

Supplement: S8 Fig — A–MS ion image of nearly isobaric PE-adduct species [PE (39:5) + H]+ and [PE (37:2) + Na]+ (Δm = 3.1 ppm) at m/z 780.5537 ± 5 ppm. B–Signal intensity (abundance in NL; normalized level) vs mass deviation in ppm. A double peak can be observed shifted by approximately 1.5 ppm and 2.8 ppm. C–MS ion signal at m/z 780.55254 ± 0.2 ppm showing an increased signal intensity on the worm surface assigned to protonated PE (39:5). D–MS ion at m/z 780.55151 ± 0.2 ppm assigned to PE (37:2) as sodium adduct. By hierarchical clustering, the signal at m/z 780.5537 was determined to be more abundant in the worm body compared to tegumental surface (see Fig 3). The signal was assigned to PE (37:2) as sodiated molecule. The protonated species of PE (39:5), however, was classified as unspecific. The fluctuating signal intensity of the surface measurements putatively led to unspecific classification. This example thus verifies the accuracy and correctness of HC-based classification. (TIF) [file pntd.0008145.s012.tif]

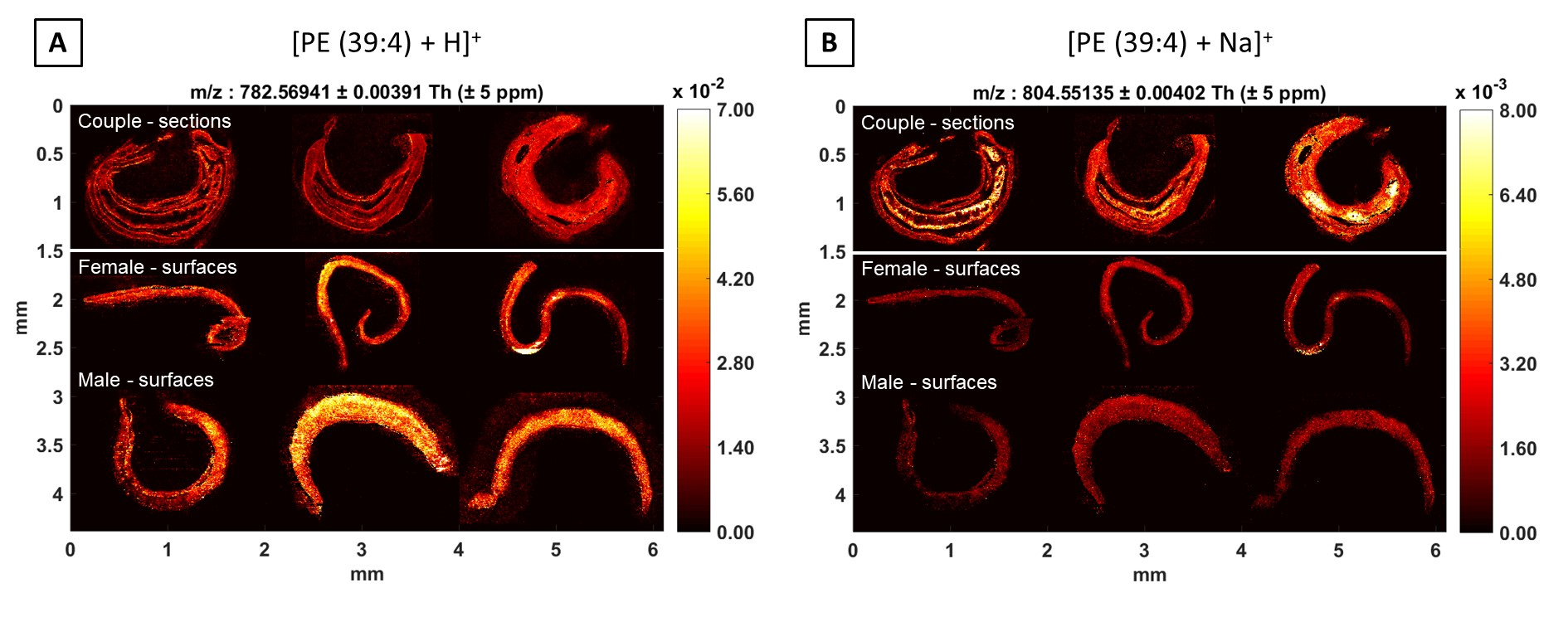

Supplement: S9 Fig — A—Distribution of m/z 782.5694 assigned to [PE (39:4) + H]+. B–distribution of m/z 804.5514 assigned to [PE (39:4) + Na]+. This difference in distribution could be explained by different concentrations of salt in tegument and inner tissue or by isobaric interferences that were not contained in the LC-MS/MS-database. (TIF) [file pntd.0008145.s013.tif]
